# A reference profile-free deconvolution method to infer cancer cell-intrinsic subtypes and tumor-type-specific stromal profiles

**DOI:** 10.1186/s13073-020-0720-0

**Published:** 2020-02-28

**Authors:** Li Wang, Robert P. Sebra, John P. Sfakianos, Kimaada Allette, Wenhui Wang, Seungyeul Yoo, Nina Bhardwaj, Eric E. Schadt, Xin Yao, Matthew D. Galsky, Jun Zhu

**Affiliations:** 1 Icahn Institute for Genomics and Multiscale Biology, Icahn School of Medicine at Mount Sinai, New York, NY 10029 USA; 2 Department of Genetics and Genomic Sciences, Icahn School of Medicine at Mount Sinai, New York, NY 10029 USA; 3 Sema4, a Mount Sinai venture, Stamford, CT 06902 USA; 4 Department of Urology, Icahn School of Medicine at Mount Sinai, New York, NY 10029 USA; 5 Department of Medicine, Icahn School of Medicine at Mount Sinai, New York, NY 10029 USA; 6 Tisch Cancer Institute, Icahn School of Medicine at Mount Sinai, New York, NY 10029 USA; 7 Department of Genitourinary Oncology, Tianjin Medical University Cancer Institute and Hospital, National Clinical Research Center for Cancer, Key Laboratory of Cancer Prevention and Therapy, Tianjin, China

**Keywords:** Deconvolution, Clustering, Bulk tumor profiling

## Abstract

**Background:**

Patient stratification based on molecular subtypes is an important strategy for cancer precision medicine. Deriving clinically informative cancer molecular subtypes from transcriptomic data generated on whole tumor tissue samples is a non-trivial task, especially given the various non-cancer cellular elements intertwined with cancer cells in the tumor microenvironment.

**Methods:**

We developed a computational deconvolution method, DeClust, that stratifies patients into subtypes based on cancer cell-intrinsic signals identified by distinguishing cancer-type-specific signals from non-cancer signals in bulk tumor transcriptomic data. DeClust differs from most existing methods by directly incorporating molecular subtyping of solid tumors into the deconvolution process and outputting molecular subtype-specific tumor reference profiles for the cohort rather than individual tumor profiles. In addition, DeClust does not require reference expression profiles or signature matrices as inputs and estimates cancer-type-specific microenvironment signals from bulk tumor transcriptomic data.

**Results:**

DeClust was evaluated on both simulated data and 13 solid tumor datasets from The Cancer Genome Atlas (TCGA). DeClust performed among the best, relative to existing methods, for estimation of cellular composition. Compared to molecular subtypes reported by TCGA or other similar approaches, the subtypes generated by DeClust had higher correlations with cancer-intrinsic genomic alterations (e.g., somatic mutations and copy number variations) and lower correlations with tumor purity. While DeClust-identified subtypes were not more significantly associated with survival in general, DeClust identified a poor prognosis subtype of clear cell renal cancer, papillary renal cancer, and lung adenocarcinoma, all of which were characterized by CDKN2A deletions. As a reference profile-free deconvolution method, the tumor-type-specific stromal profiles and cancer cell-intrinsic subtypes generated by DeClust were supported by single-cell RNA sequencing data.

**Conclusions:**

DeClust is a useful tool for cancer cell-intrinsic molecular subtyping of solid tumors. DeClust subtypes, together with the tumor-type-specific stromal profiles generated by this pan-cancer study, may lead to mechanistic and clinical insights across multiple tumor types.

## Background

Molecular subtyping of tumors based on transcriptomic data generated from tumor tissue is increasingly being used as a means of categorizing biology shared across patients’ tumors and informing prognosis and response to treatment [1, 2]. Many computational methods have been developed for such purposes [3, 4]. However, solid tumor tissues are a mixture of cancer, immune, and non-hematopoietic stromal cells. Importantly, aside from intra- and inter-tumor type variability among these cellular components, different sample preparation and profiling techniques may result in biologically irrelevant variations in the quality and quantity of each cellular compartment. Thus, deriving cancer cell-intrinsic subtypes by dissecting the contribution of cancer cells from other elements in the tumor microenvironment may generate better biomarkers and mechanistic insights.

Several groups including ours have developed computational deconvolution algorithms to dissect cellular compartments from bulk tumor transcriptomic data [5–13]. However, despite the potential clinical importance of molecular subtyping of tumors, existing deconvolution methods suffer from three key limitations: (1) Existing methods do not incorporate cancer subtypes into the modeling process nor do they output cancer cell-intrinsic subtypes directly. Although for some of the methods [8], cancer cell subtypes could theoretically be generated by downstream analyses following initial deconvolution [10], the accuracy of such a strategy has not been systematically evaluated. (2) Existing methods rely on input of reference profiles for the different cellular compartments [5, 8, 12, 13], the choice of which can substantially impact the accuracy of the results [10]. However, appropriate input reference profiles are rarely available for any individual tumor type and the use of fixed profiles across tumor types is suboptimal due to cellular heterogeneity across tumor types. (3) Existing methods focus on estimating cell compartment fractions [8–10], but do not output tumor-type- or tissue-specific gene expression values for each cellular compartment. The estimated expression values of all genes for cancer cells corresponding to a molecular cancer subtype or for the stromal compartment of a particular tissue can be critical for achieving a better understanding of patient prognosis and drug response.

Here, we present a computational method, DeClust, for modeling bulk tumor tissue transcriptomic data that simultaneously delivers a reference profile-free deconvolution of bulk tumor gene expression data into cancer, immune, and stromal cellular compartments, as well as a cancer cell-intrinsic clustering of cancer samples to uncover molecular cancer subtypes. By modeling cancer transcriptomic data as a mixture of three cellular compartments (cancer, immune, and stromal compartments) and stratifying samples into multiple subtypes based on the cancer cellular compartment, our algorithm outputs the cancer cell-intrinsic subtype for each sample as well as the fraction and estimated reference expression profile for each cellular compartment. The output cancer cell-intrinsic subtype reference profiles are for the dataset, not for individuals as ISOpure [14] and DeMixT [15] do.

Using simulated data, we compared DeClust to existing methods in widespread use. DeClust achieved superior accuracy in clustering samples into subtypes. We then applied DeClust to a pan-cancer dataset comprised of 13 tumor types from The Cancer Genome Atlas (TCGA) [16]. From the DeClust outputs generated on this dataset, we show that (1) stromal compartments are associated with patient survival in a tumor-type-specific manner, (2) the subtypes identified by DeClust were associated with enhanced cancer cell-intrinsic characteristics than those based on the existing TCGA molecular subtypes or alternative strategies, and (3) while DeClust identified subtypes were not more significantly associated with survival in general, a poor prognosis subtype relevant to several tumor types was identified by DeClust that was not evident based on the existing TCGA molecular subtypes. Finally, we generated single-cell RNA sequencing data and assembled publicly available single-cell data to validate the cell-type-specific compartment profiles and cancer-intrinsic subtypes inferred by DeClust.

## Methods

### Data sources

The RNAseq gene expression data, copy number data, methylation data, mutation data, and survival data for the 13 TCGA [16] datasets were downloaded from Broad Institute Firehose (https://gdac.broadinstitute.org/) (2016_01_28). The list of genes that are significantly mutated in each dataset assessed by Mutsig [17] and the genes that are significantly amplified/deleted estimated by GIST [18] were also downloaded directly from Firehose (206_01_28, Aggregate_AnalysisFeatures). For each dataset, genes with a standard deviation less than 0.5 (log scale) were filtered out. The Cancer Cell Line Encyclopedia (CCLE) [19] gene expression data were downloaded from Broad Institute (https://broadinstitute.org/ccle/). The non-TCGA tumor expression datasets used in validation were downloaded from GEO [20] database according to the GSE number as provided in the manuscript. The scRNAseq data of two BLCA tumors that were generated in the study are deposited into GEO database as GSE130001 [21].

### TCGA subtypes

The sample clustering results based on gene expression profiles were downloaded from the supplementary tables of the original TCGA paper when available. If not available in the original paper, we used the clustering results from Firehose (by cNMF [3] method). For those new samples added after the initial TCGA publications, we applied the PAM [22] algorithm as implemented in R package *pamr* to assign their subtypes. In particular, we trained the PAM model using the subset of samples with TCGA subtyping available and then predicted the TCGA subtype for each newly added sample using the trained PAM models.

### Other deconvolution methods in the analysis of TCGA datasets

EPIC, quanTIseq, and the absolute version of CIBERSORT were applied to the 13 TCGA datasets using R package *immunedeconv* (V2.0.0) [23] with default parameters and input signature matrix. ISOpure was run through the R package *ISOpureR* (V1.1.3) downloaded from https://cran.r-project.org/web/packages/ISOpureR/index.html. The algorithm ISOpure requires both normal tissue expression profiles and tumor expression profiles as inputs. We used the normal tissue expression data provided by TCGA for each cancer type. TCGA OV dataset was not analyzed by ISOpure since there was no normal tissue data available for OV in TCGA. The ISOpure program ran for TCGA BRCA dataset did not finish after 14 days using the processor Intel 8168 (24C, 2.7 GHz) with 4G memory. We thus only assessed the performance of ISOpure across 11 out of the 13 TCGA datasets.

For EPIC, the fraction of the immune compartment was calculated by summing up the five immune cell frequencies estimated by the algorithm (B cells, CD4 T cells, CD8 T cells, macrophages and NK cells). The fraction of the stromal compartment was the sum of two stromal cell frequencies output by EPIC (CAF and endothelial cells). The tumor purity was equivalent to the fraction of “other cells” estimated by EPIC. For the absolute version of CIBERSORT, the fraction of the immune compartment was calculated by the sum of the 22 immune cell fractions estimated by the absolute version of the algorithm. The tumor purity was 1 minus the fraction of the immune compartment. For quanTIseq, the fraction of the immune compartment was calculated by the sum of 10 immune cell fractions output from the algorithm and the purity to the fraction of “other cells” by quanTIseq.

The two-step strategy to obtain cancer cell-intrinsic subtypes from EPIC and CIBERSORT was similar to that used in the simulation study. In the first step, as shown in the following formula, we estimated the cancer cell expression profile ${E}_{i,j}^{\mathrm{cancer}}$ for each sample by subtracting from the mixed expression profile the contribution from each immune or stromal cell types.

${o}_{i,j}^{\mathrm{mix}}$ denotes the mixed expression of gene *i* in sample *j* (in the original scale, not log-transformed). $\kern0.5em {refE}_i^{\mathrm{immune}\_k}$ and $\kern0.5em {refE}_i^{\mathrm{stromal}\_k}$ (also in the original scale) denote the reference expression of gene *i* for immune cell type *k* and stromal cell type *k*, respectively. ${f}_j^{\mathrm{immune}\_k}$ and ${f}_j^{\mathrm{stromal}\_k}$ represents the corresponding cell type frequency for sample *j*. The reference profiles for different cell types were the ones used in the corresponding deconvolution algorithm, i.e., EPIC or CIBERSORT, and the cell type frequencies were estimated by the deconvolution algorithm. The algorithm quanTIseq was not assessed for the two-step clustering strategy since its reference expression profiles were not available (only the signature matrix was available). It is of note that the original reference expression profiles may not be in the same scale as the observed mixed TCGA expression profiles; we thus multiplied the reference expression matrix by a scaling factor to make the median of the reference expression matrix to be the same as that of the mixed expression matrix before applying the above formula. In the second step, *K*-means clustering was applied to ${E}_{i,j}^{\mathrm{cancer}}$. Since *K*-means clustering is sensitive to the scale of the data, we quantile-normalized the obtained cancer cell expression profile before applying *K*-means; we found this step improved the clustering results.

### Pathway analysis

In the pan-cancer analysis of tissue/subtype-specific expression profiles derived based on DeClust, pathway scores were calculated using the ssGSEA method as implemented in the R package *GSVA* [24]. To identify pathways significantly up/downregulated in the stromal profile of a particular TCGA dataset as compared to that of other datasets, we first carried out a gene-wise Z-transformation across the 13 stromal profiles. Then within each profile, we assessed whether a particular gene set showed a significantly higher/lower expression value by Wilcoxon rank-sum test.

### Survival analysis

Cox-regression model and the log rank test were used to assess the significance of associations between survival and cancer subtypes as well as samples with high/low stromal proportion. To define samples with high/low immune/stromal proportion, we first split the samples into three groups of equal size according to the 1/3 and 2/3 quantile value of the immune/stromal proportion and then removed the middle group from the analysis.

### Validation in non-TCGA datasets

Multiple approaches to assign each sample in the non-TCGA dataset to DeClust subtypes were considered. For example, we could apply DeClust to each non-TCGA dataset and generate new subtypes de novo. Alternatively, we could train a classifier using the TCGA expression profiles and the DeClust subtype annotation and then predict the class label for each sample in the validation dataset. We used this latter strategy since it was less time-consuming, imposed no sample size requirement for the validation dataset, and allowed us to take advantage of existing classification algorithms. Specifically, we used the PAM method to train classifiers based on DeClust subtypes of TCGA and then applied them to the corresponding validation datasets. As a fair comparison, we used a similar strategy to assign the original TCGA subtypes to each sample in the validation datasets as well.

To estimate immune/stromal cell proportions in the non-TCGA datasets, we again did not generate those de novo using DeClust, but rather used a less time-consuming strategy. We defined tissue/cancer-type-specific stromal genes as those whose expression showed a high correlation with the stromal proportions estimated by DeClust (Spearman’s CC > 0.8). Based on these tissue/cancer-type-specific stromal gene sets, we then estimated the stromal proportions in the non-TCGA dataset using ssGSEA similar to the approach employed by ESTIMATE. The immune proportions were estimated similarly.

### Single-cell RNA sequencing of two bladder cancer specimens

The genomic study was approved by the Icahn School of Medicine Institutional Review Board (#10-1180). The fresh muscle-invasive bladder tumor samples were minced to 1–2-mm chunks via mechanical dissociation. The samples were subsequently undergone mechanical/chemical dissociation using the Mintenyi Biotec Gentlemacs 130-096-427 Octo Dissociator at the h_2 dissociation program. Upon completion of the dissociation, the dissociated tumor and buffer was filtered to single-cell suspension, using the 70-μm Gentelmacs filter. The suspension was then spun down and the dissociation buffer was removed, followed by the addition of 1% ACK lysis buffer for RBC lysis for 1 min. The RBC lysis buffer was then diluted with DBPS, and the bladder cancer cells were then spun down at 200–300 G. After a final wash, the supernatant was discarded, and the pellet was resuspended in cold FACS buffer (DPBS containing 2% BSA and 2 mM EDTA). Bladder cancer single cells were analyzed by fluorescence-activated cell sorting (FACS) to determine phenotype. In brief, cells were stained with fixable viability dye blue to ascertain viability (ThermoFisher) and BV510-labeled anti-CD45 (BioLegend). For the sort, cells were gated on live singlets followed by the CD45 makers. All CD45− were collected.

Prior to the preparation of single-cell sequencing libraries, the cell density was determined using a hemocytometer using a mixture of trypan blue and cell suspension was examined under the microscope at low magnification using the Evos Cell Imaging System-digital inverted microscope. This assessment of cell viability ensured successful preparation. The single-cell Chromium chip loading, gel emulsion (GEM) generation and barcoding, post GEM_RT and cDNA amplification, and library construction were performed according to the Chromium™ Single Cell 3′ Protocol - Chemistry version 2 (10X Genomics). For GEM generation, an input of > 10,000 cells in total was targeted for each sample, with a target cell recovery goal of 2000+ cells. For the cDNA amplification reaction, we used 12 cycles during incubation for a targeted cell recovery of interest (2000–6000 cells). Quantification of the synthesized cDNA was evaluated using Qubit (Qubit dsDNA HS Assay Kit, Thermo Fisher), and Agilent cDNA High Sensitivity Kit, following the manufacturers’ instructions. During library construction, the sample index PCR incubation was performed according to the protocol’s recommendation. Quantification of the constructed libraries was evaluated using Qubit (Qubit dsDNA HS Assay Kit, Thermo Fisher), Agilent cDNA High Sensitivity Kit, and Kapa DNA Quantification Kit for Illumina platforms, following the manufacturers’ instructions. Each sample was then sequenced using the Illumina HiSeq 2500 platform leveraging one lane per sample to assure 50,000 reads or more per cell for expression profiling. Cell Ranger pipeline from 10X genomics (Pleasanton, CA) was used to align reads and generate gene-cell matrices. The scRNAseq data are deposited into GEO database as GSE130001 [21].

### Analysis of single-cell RNA sequencing data

The scRNAseq read count data for three ccRCC samples and one pRCC sample were downloaded from the supplementary table of the previous study [25]. We selected only those cells passing the original quality control and showing zero CD45 read count. Note that some immune cells with zero CD45 read counts still remained in our analysis, likely reflecting dropout events [26] or sampling artifacts given limited sequencing depth. Since one of the ccRCC samples has only 29 cells left, it was removed from further analyses. There were a total of 6781, 757 and 649 cells for the two ccRCC samples and one pRCC sample, respectively. For the two BLCA specimens we sequenced (GSE130001 [21]), there were 3422 and 588 cells after quality control for the two samples. The median number of detected genes per cell was 2560.

We used *Seurat* [27] to analyze the scRNAseq data. Briefly, after the read count data was normalized, the most variable genes were selected. Then the effect of unique molecular identifier (UMI) count and percentage of mitochondria per cell was regressed out, followed by dimension reduction using PCA. Finally, the cells were clustered using the *K*-nearest neighbors graph-based methods as implemented in *Seurat* and then annotated the cell clusters based on cell-type-specific markers [25, 28].

### DeClust algorithm

This section is organized as follows. We first lay out the general framework of the DeClust algorithm. Then we provide a detailed description of each step. Finally, we explain how certain parameters were determined including the number of subtypes.

A bulk tissue expression profile can be decomposed according to tissue/subtype-specific expression profiles and the sample-specific abundance of each component as in formula (1):

1 $$ {\left.{\exp}^{o_{i,j}^{\mathrm{mix}}}\right|}_{\mathrm{subtyp}\mathrm{e}(j)=\mathrm{subtyp}{\mathrm{e}}_k}\kern0.5em \sim {\exp}^{E_i^{\mathrm{subtyp}{\mathrm{e}}_k}}\times {f}_j^{\mathrm{cancer}}+{\exp}^{E_i^{\mathrm{immune}}}\times {f}_j^{\mathrm{immune}}+{\exp}^{E_i^{\mathrm{stromal}}}\times {f}_j^{\mathrm{stromal}} $$

where ${o}_{i,j}^{\mathrm{mix}}$ denotes the observed mixed expression (log-transformed) for gene *i* and sample *j*; ${f}_j^{\mathrm{cancer}},{f}_j^{\mathrm{immune}}$, and ${f}_j^{\mathrm{stromal}}$ represent the cancer, immune, and stromal proportion for sample *j*, respectively; ${E}_i^{\mathrm{immune}}$, ${E}_i^{\mathrm{stromal}}$, and ${E}_i^{\mathrm{subtyp}{\mathrm{e}}_k}$ denote the expected expression value (log-transformed) of gene *i* in immune cell, stromal cell, and cancer cell of subtype *k*, respectively; and subtype(*j*) = subtype_*k*_ denotes sample *j* belonging to cancer subtype *k*. Note the linear combination of expression values across the different components was carried out in the original scale (before log-transformation).

There are three groups of unknown variables in formula (1): the sample-wise variables of the different cell compartments ${f}_j^{\mathrm{cancer}},{f}_j^{\mathrm{immune}}$, and ${f}_j^{\mathrm{stromal}}$ (group A), the size of which equals to three times of the sample size; the sample-wise variables of subtype assignment subtype(*j*) (group B), the size of which equals to the sample size; and the gene-wise variables of the expected expression values ${E}_i^{\mathrm{immune}}$, ${E}_i^{\mathrm{stromal}}$, and ${E}_i^{\mathrm{subtyp}{\mathrm{e}}_k}$ (group C), the size of which equals to the number of genes multiplied by (2 + the number of cancer subtypes). The known variables in formula (1) are the observed expression levels of the mixture ${o}_{i,j}^{\mathrm{mix}}$, the size of which equals to the gene count multiplied by the sample size. We estimated the above three groups of unknown variables by minimizing the mean square error (MSE) of formula (1), i.e., the difference between observed and reconstructed expression values as shown in formula (2):

2 $$ \mathrm{MSE}=\sum \limits_{i,j}{\left({o}_{i,j}^{\mathrm{mix}}-\log \left(\ {\mathit{\exp}}^{E_i^{\mathrm{subtyp}{\mathrm{e}}_k}}\times {f}_j^{\mathrm{cancer}}+{\mathit{\exp}}^{E_i^{\mathrm{immune}}}\times {f}_j^{\mathrm{immune}}+{\mathit{\exp}}^{E_i^{\mathrm{stromal}}}\times {f}_j^{\mathrm{stromal}}\right)\right)}^2 $$

Note the MSE is calculated after log-transformation assuming the gene expression follows log-normal distribution. Since the count of known variables in formula (1) is significantly larger than that of unknown variables for TCGA datasets where the sample number is generally around 200, optimal mathematical solution is expected.

To find the parameter estimates that minimize the MSE, we employed a two-layer (an inner and outer layer) optimization procedure to iteratively optimize the three groups of unknown variables. In the outer layer, we iteratively optimized group A variables, given estimates for parameters associated with the group B and C variables. In the inner layer, given the group A variables, we iteratively optimized between the group B and C variables. The initial value of cell component (group A) was derived based on known marker gene expression values, and the initial sample subtype (group B) was obtained from *K*-means clustering of the residual expression. We used gradient descent method “L-BFGS-B” implemented in R function optimx() to find the optimal solution in each step. The step-by-step process of the DeClust algorithms is detailed below, and the schematic illustration can be found in Additional file 1: Figure S1.

- Step 0: In this step, we set the initial values of cell components. Assuming the immune and stromal cell abundance is proportional to its corresponding marker expression, the cell component for each sample can be calculated as follows:

3 $$ {\displaystyle \begin{array}{c}{f}_j^{\mathrm{immune}}={\mathit{\exp}}^{o_{\mathrm{immune}\mathrm{Markers},j}^{\mathrm{mix}}}\times {C}_{\mathrm{immune}}\ \\ {}{f}_j^{\mathrm{stromal}}={\mathit{\exp}}^{o_{\mathrm{stromal}\mathrm{Markers},j}^{\mathrm{mix}}}\times {C}_{\mathrm{stromal}}\\ {}{f}_j^{\mathrm{cancer}}=1-{f}_j^{\mathrm{immune}}-{f}_j^{\mathrm{stromal}}\end{array}} $$

where ${o}_{\mathrm{immuneMarkers},j}^{\mathrm{mix}}$ and ${o}_{\mathrm{stromalMarkers},j}^{\mathrm{mix}}$ are the mean expression values of immune and stromal markers for sample *j* (averaged in log scale), and *C*_immune_ and *C*_stromal_ are two constants (how to determine the value of the two constants are described later).

- Step 1: Given ${f}_j^{\mathrm{cancer}},{f}_j^{\mathrm{immune}},\kern0.5em \mathrm{and}\ {f}_j^{\mathrm{stromal}}$ for each sample, we optimize ${E}_i^{\mathrm{subtyp}{\mathrm{e}}_k}$, ${E}_i^{\mathrm{immune}}$, and ${E}_i^{\mathrm{stromal}}$ for each gene and subtype assignment for each sample subtyp(*j*) through an inner layer of iterative optimization (step 1.0 to step 1.3):
  Step 1.0: In this step, we set the initial subtype assignment for each sample as the following. We first assume there is only one cancer subtype, i.e., subtyp(*j*) =1 for each sample *j*; formula (1) is then simplified as

4 $$ \kern1.5em {\mathit{\exp}}^{o_{i,j}^{\mathrm{mix}}}\sim {\mathit{\exp}}^{E_i^{\mathrm{subtyp}{\mathrm{e}}_1}}\times {f}_j^{\mathrm{cancer}}+{\mathit{\exp}}^{E_i^{\mathrm{immune}}}\times {f}_j^{\mathrm{immune}}+{\mathit{\exp}}^{E_i^{\mathrm{stromal}}}\times {f}_j^{\mathrm{stromal}} $$

By minimizing MSE, we obtained the optimal ${E}_i^{\mathrm{subtyp}{\mathrm{e}}_1},{E}_i^{\mathrm{immune}},\mathrm{and}\ {E}_i^{\mathrm{stromal}}$ for each gene *i*. We then calculated the residual expression *r*_*i*, *j*_ for gene *i* and sample *j* as the difference between the observed and reconstructed expression value (in log-transformed scale):

5 $$ {r}_{i,j}={o}_{i,j}^{\mathrm{mix}}-\log \left({\exp}^{E_i^{\mathrm{subtyp}{\mathrm{e}}_1}}\times {f}_j^{\mathrm{cancer}}+{\mathit{\exp}}^{E_i^{\mathrm{immune}}}\times {f}_j^{\mathrm{immune}}+{\mathit{\exp}}^{E_i^{\mathrm{stromal}}}\times {f}_j^{\mathrm{stromal}}\right) $$

The initial subtype assignment is obtained by *K*-means clustering of *r*_*i*, *j*_ given the number of subtypes (how to choose the optimal number of subtypes is described later).

- Step 1.1: Given subtype assignment for each sample subtyp(*j*), we optimize ${E}_i^{\mathrm{subtyp}{\mathrm{e}}_k}$, ${E}_i^{\mathrm{immune}}$, and ${E}_i^{\mathrm{stromal}}$ for each gene by minimizing MSE in formula (2).
- Step 1.2: Given ${E}_i^{\mathrm{subtyp}{\mathrm{e}}_k}$, ${E}_i^{\mathrm{immune}}$, and ${E}_i^{\mathrm{stromal}}$ for each gene, we optimize subtype assignment for each sample subtyp(*j*) to minimize MSE in formula (2).
- Step 1.3: Repeat step 1.1 and step 1.2 until converge (end of the inner layer of iterative optimization).
- Step 2: Given ${E}_i^{\mathrm{subtyp}{\mathrm{e}}_k}$, ${E}_i^{\mathrm{immune}}$, ${E}_i^{\mathrm{stromal}}$, and subtyp(*j*) that are optimized through the inner layer of iterative optimization, we optimize ${f}_j^{\mathrm{cancer}},{f}_j^{\mathrm{immune}},\mathrm{and}\ {f}_j^{\mathrm{stromal}}$ for each sample by minimizing MSE in formula (2).
- Step 3: Repeat step 1 and step 2 until converge (end of the outer layer of iterative optimization).

It is of note that there are three unknown parameters in the above algorithm that need to be determined. Two of them are *C*_immune_ and *C*_stromal_, which are used to transform marker gene expression to tissue proportions. Here is how we determine them: for a given number of subtype, we used the grid search to find the optimal pair of *C*_immune_ and *C*_stromal_. Because the tissue fraction is between 0 and 1, the possible value of *C*_immune_ and *C*_stromal_ forms a constrained two-dimensional space. We then divided the space into a 10 by 10 grid. For each pair of *C*_immune_ and *C*_stromal_ in the grid, we run step 1 and step 2 above to obtain MSE (we did not use iteration in this case to save computation time). The pair of *C*_immune_ and *C*_stromal_ which gives the smallest MSE was chosen as optimal.

The third parameter to be determined is the optimal number of subtypes. We used BIC curves as the guidance to select the optimal number of subtypes. Specifically, we iterated the subtype number from 1 to 10 and calculated MSE and BIC (log (MSE) × sampleNum+log (sampleNum) × subtypeNum) for each subtype number. Two strategies were used to select the best subtype number: (1) the one giving the minimum BIC and (2) the one at the elbow point of the BIC curve. When the two criteria gave two different results, the medium of the two was chosen as the final one.

### Simulations

In the simulation study, we used bladder cancer as an example. Following formula (1), we simulated mixed BLCA expression profiles with reference (expected) expression profiles for BLCA cancer cells of different subtypes, immune cells and stromal cells as detailed below.

For simplicity, we assumed there were three BLCA subtypes, i.e., luminal, basal, and neuronal. The reference expression profile for each subtype was represented by that of a BLCA cell line of the corresponding subtype. To choose the best representative cell line, we mapped each BLCA TCGA sample to the most similar CCLE BLCA cell lines (highest Spearman’s correlation). For example, the cell line that was mapped by the most TCGA samples of luminal subtypes (the original TCGA subtyping was used here for convenience) was chosen to represent the luminal subtype. As a result, the expression profiles of SW780, BFTC950, and KU1919 cell lines in CCLE were chosen as the reference cancer cell profile for luminal, basal, and neuronal subtype, respectively.

The immune components in the tumor are a mixture of different immune cells. Thus, we reconstructed the reference immune profile using the expression profile for each type of immune cells (downloaded from CIBERSORT [8]) and the fraction of each immune cell type (downloaded from the immune landscape [29]). Although DeClust does not consider subtype-specific immune profile, we did consider the possible subtype-specific difference in simulating the data, i.e., the proportion of immune cell types might differ for different subtypes. We thus constructed reference immune profile for each subtype separately, using the mean fraction of each immune cell type within that particular subtype. The reference immune profile for each subtype derived in this way is very similar to each other (Spearman’s CC > 0.99), much higher than the correlation between cancer profiles (Spearman’s CC = 0.86, 0.87 and 0.88). This supports the hypothesis that the majority of inter-tumor heterogeneity comes from cancer cells, based on which subtype was designed.

The stromal component was simulated here by expression profile of cancer-associated fibroblast (CAF). Since there is no BLCA-specific CAF data available, we used CAF data from breast cancer (GSE37614 [24]) as a replacement. Similar to immune profile, we allowed subtype-specific difference in stromal profile by using CAF associated with ER, Her2 and TNBC subtype in breast cancer to represent the stromal profile of the three BLCA subtypes respectively. Again, the similarity between each pair of the three stromal profiles (Spearman’s CC = 0.99, 0.96 and 0.96) is much higher than similarities of cancer cells of different subtypes.

To simulate the mixed expression profile for a subtype, we used the reference expression profiles of each component for that particular subtype as described above. We then randomly sampled the fraction for each component according to their distribution in that subtype in the TCGA data (downloaded from the immune landscape [29]). The mixed expression profile was then calculated according to formula (1). Finally, random noise was added to the log scale of the mixed expression value with noise following either a log normal distribution (~N(0,noiselevel) at a given noise level) or a negative binomial distribution with the dispersion parameter within the range that matched the gene variation simulated under a log-normal distribution. We simulated datasets at different sample sizes (100, 200, and 300) and different noise levels. For a given sample size, the number of samples simulated for each cancer subtype follows the subtype proportion in the TCGA BLCA dataset. We simulated 20 datasets at each combination of sample size and noise level.

### Comparison with other methods in simulations

#### CIBERSORT

The reference expression profiles for the three cellular compartments and the three subtypes (9 profiles in total) were input to CIBERSORT’s feature selection program to construct the feature matrix (each of the three subtypes was treated as a replicate of the cellular compartment). The feature matrix was then used as an input for the CIBERSORT deconvolution algorithm.

#### *K*-means clustering

Subtyping the simulated data by *k*-means clustering was performed in two ways. One way was that *k*-means clustering was applied to the data directly without considering heterogeneity of the samples. Alternatively, the subtyping followed a two-step strategy noted as CIBERSORT-k-means: fractions of cell components, ${f}_j^{\mathrm{cancer}},{f}_j^{\mathrm{immune}}$, and ${f}_j^{\mathrm{stromal}}$ were estimated by CIBERSORT as described above, and the cancer profile ${E}_{i,j}^{\mathrm{cancer}}$ for gene *i* in sample *j* was calculated as follows:

$$ {E}_{i,j}^{\mathrm{cancer}}=\frac{o_{i,j}^{\mathrm{mix}}-{\mathrm{ref}E}_i^{\mathrm{immune}}\times {f}_j^{\mathrm{immune}}-\mathrm{ref}{E}_i^{\mathrm{stromal}}\times {f}_j^{\mathrm{stromal}}}{f_j^{\mathrm{cancer}}}, $$

where ${refE}_i^{\mathrm{immune}}$ and $\mathrm{ref}{E}_i^{\mathrm{stromal}}$ were the reference expression profiles used in simulation (median of the three subtypes). Then, *K*-means clustering was applied to the estimated cancer profile ${E}_{i,j}^{\mathrm{cancer}}$.

## Results

### Overview of the DeClust algorithm and evaluation strategy

The general framework for most transcriptomic deconvolution algorithms involves reconstruction of bulk tissue gene expression data from gene expression profiles for individual cell compartments [30]. Given the appropriate reference gene expression profiles, the fraction of each compartment is estimated by minimizing reconstruction errors [5, 7]. Alternatively, if the fractions of the different cell compartments are known, reference gene expression profiles can be directly estimated [31, 32]. However, in most cases, neither accurate cell compartment fractions nor proper reference expression profiles are available. Thus, DeClust employs a multi-layer optimization strategy to infer this information from the available data.

**Fig. 1:**
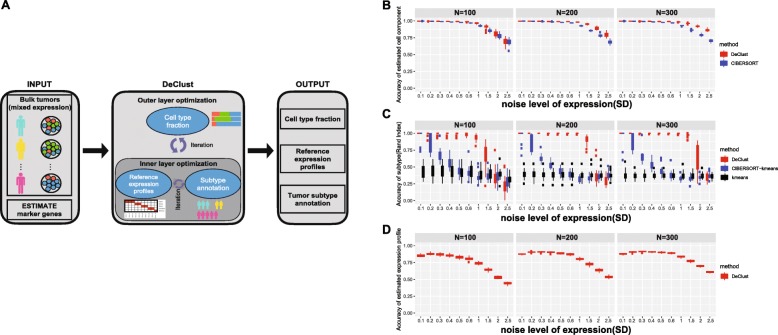
Flow chart of the DeClust algorithm (**a**) and the simulation results (**b**–**d**). The accuracy of the estimated cell compartment fraction in **b** was calculated via the correlation between simulated and estimated cell frequency profiles (average for the three components). The accuracy of the estimated expression profiles in **d** was calculated by correlation between simulated and estimated expression profiles. Specifically, the average correlations over the cancer, immune, and stromal profiles are plotted at different noise levels and sample sizes. The noise levels represent the standard deviation of the noise added to the simulated mixed expression data under the log-normal distribution (see the “Methods” section)

DeClust begins by generating initial estimates of the immune and stromal compartment fractions based on the expression levels of well-known marker genes (those used by ESTIMATE [6]). The cancer compartment fraction is then computed as the difference between the whole and the immune and stromal compartment fractions. Given these initial estimates of the compartment fractions, DeClust, in an iterative fashion (depicted as the inner optimization layer of the DeClust algorithm in Fig. 1a), estimates the intertumoral heterogeneity representing different molecular states by considering each sample as having come from a different cancer subtype. With each sample clustered into a molecular subtype supported by the expression data, DeClust then estimates the expected expression values for each gene for each inferred cancer subtype and each non-cancer compartment. Given the cancer subtype assignments and expected gene expression levels for each cancer subtype (representing the reference profiles for each cancer subtype) generated by the inner optimization layer, the DeClust algorithm generates new estimates for the cell compartment fractions in a second iterative process that is similar to matrix factorization [33] (depicted as the outer optimization layer in Fig. 1a). Whereas the inner optimization layer addresses intertumoral heterogeneity across subtype-specific molecular states, the outer optimization layer addresses intratumoral heterogeneity across the different cellular compartments. By iterating between the inner and outer optimization layers, the DeClust algorithm aims to find the following estimates that might minimize the reconstruction errors: (1) the proportion of cancer, immune, and stromal cells in each sample; (2) the cancer cell-intrinsic subtype to which each sample belongs; and (3) the inferred gene expression profiles for each cancer subtype as well as for the immune and stromal compartments, which collectively serve as the output for each run.

To evaluate the performance and accuracy of DeClust, we applied it to simulated datasets and to a pan-cancer dataset from TCGA. The simulated dataset was employed to directly compare the performance of DeClust to other deconvolution approaches in widespread use. The metrics to assess performance center on demonstrating the accuracy of DeClust across its primary outputs (cell compartment fractions, classification of samples to subtypes, and compartment-wise expression values across all genes) under different sample size and noise conditions, by comparing the estimates generated by DeClust to the known values used to generate the simulated datasets. For the pan-cancer dataset, we again focused on the accuracy of the primary outputs, but because ground truth in the pan-cancer dataset is not known, we examined how DeClust outputs compared to other approaches with regard to patient survival, known drive mutations, tumor purity, and cell compartment-specific gene expression (see Additional file 1: Figure S2 for an overview of our study workflow).

### Assessment of DeClust based on simulated datasets

We simulated bulk tumor expression datasets where expression profiles for the immune, stromal, and cancer compartments; number of cancer subtypes; and cancer subtype assignment of each sample were all known (see the “Methods” section) and then applied DeClust to the simulated datasets and compared the outputs to existing methods to assess their performance based on multiple criteria.

To assess the performance and accuracy of estimating the cell compartment fractions, we compared DeClust with one of the more commonly used deconvolution methods, CIBERSORT [8]. Both DeClust and CIBERSORT achieved good accuracy and their results were broadly comparable (Fig. 1b). As expected, accuracy improved with increasing sample size and decreased as noise levels were increased in the expression data. DeClust was more robust than CIBERSORT with respect to the accuracy of the compartment fraction estimates as noise levels and sample sizes were increased. We note that the CIBERSORT results in our simulation analysis were more idealized (thus the accuracies reported here are inflated) than would be achieved in practice, given we provided the reference expression profiles used to simulate the data as input for CIBERSORT to generate its needed feature matrix. In practice, the context-specific reference expression profiles would not likely be available.

To assess the performance and accuracy of classifying samples into the most appropriate molecular subtype, we compared the DeClust subtype assignments to those generated by two related approaches: (1) ordinary *K*-means clustering applied directly to the simulated bulk tumor expression profiles and (2) a two-step strategy in which CIBERSORT was first applied to estimate the cell compartment fractions as described above, and then K-means clustering was applied to the resulting cancer cell profiles (derived for each sample by removing the immune and stromal compartments from the simulated bulk tumor gene expression data) [10] (Fig. 1c). As shown in Fig. 1c, the performance of ordinary *K*-means was the least favorable approach, even when noise levels were low, indicating that the varied immune and stromal compartment fractions adversely affected the accuracy of the sample subtyping assignments. The two-step strategy gave better results when noise levels were low, demonstrating the utility of removing the effects of the non-cancer compartments. However, as noise levels were increased, the performance of the two-step strategy deteriorated rapidly. In contrast, DeClust was much more tolerant to increasing noise levels and remained at a high level of accuracy until noise levels were much higher than those observed in the TCGA datasets (Additional file 1: Figure S3).

Finally, to assess the performance and accuracy of inferring the reference expression profiles, we computed the Pearson correlation coefficients between the profiles used in the simulations and those inferred by DeClust. The accuracy hovered around 0.8 when noise levels were within the range of those observed in the TCGA datasets (Fig. 1d).

The results across all of the DeClust outputs were similar (Additional file 1: Figure S4) when noise was simulated following a negative binomial distribution (a common assumption for RNAseq data) as opposed to the log-normal distribution (a common assumption for microarray gene expression data) assumed above. Together, these results suggest that as a reference profile-free method, DeClust achieves accuracies for cell component fraction estimations that are comparable to reference profile-based deconvolution methods. However, DeClust has the unique advantage over these methods of accurately inferring the cancer cell-intrinsic subtypes for each sample as well as the compartment-specific gene expression profiles.

### Pan-cancer analysis by DeClust

**Fig. 2:**
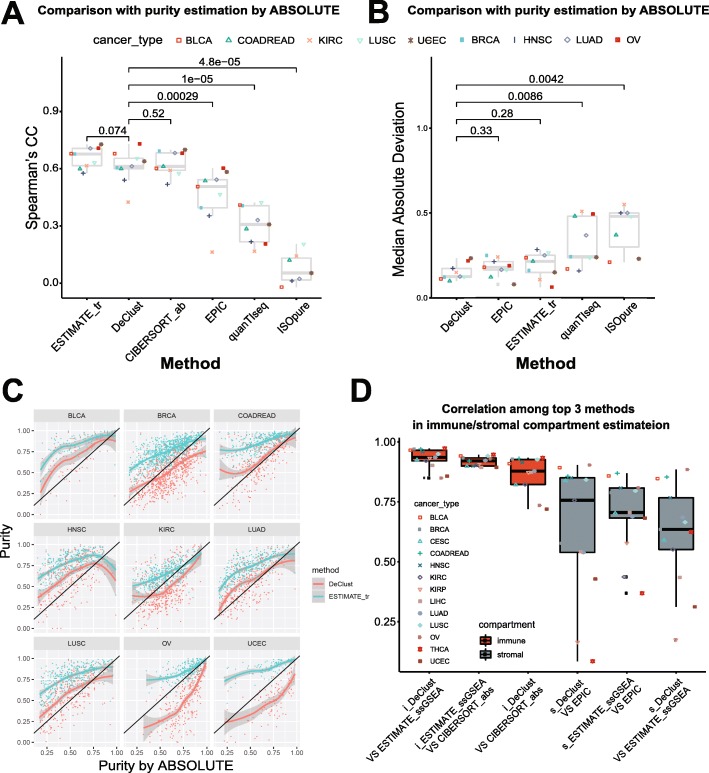
Comparison of cell fractions estimated by different methods. Comparing tumor purity (cancer cell fraction) estimation by different deconvolution methods with the one based on ABSOLUTE (treated as “ground truth”) in terms of Spearman’s correlation coefficients (**a**) or median absolute deviation (**b**). The *p* values above are the difference between DeClust and other methods according to the two-sided paired *t* test. **c** Scatter plot of tumor purity estimates by DeClust or ESTIMATE_tr against the one by ABSOLUTE for each cancer type (ESTIMATE_tr transformed ESTIMATE ssGSEA score to fit ABSOLUTE estimates.). **d** Correlation among the top 3 deconvolution methods (i.e., DeClust, ESTIMATE_ssGSEA, and CIBERSORT_abs) in estimations of immune cell fractions (red), and correlation among the top 3 deconvolution methods (i.e., DeClust, EPIC, and ESTIMATE_ssGSEA) in estimations of stromal cell fractions (gray)

We applied DeClust to 13 solid tumor datasets from TCGA and characterized cancer, immune, and stromal compartment outputs from DeClust. We also employed other available gene expression deconvolution methods, i.e., ESTIMATE [6], the absolute version of CIBERSORT [8], EPIC [34], quanTIseq [35], and ISOpure [14]. We chose these methods because their estimation allows both intra-sample comparisons between cell types and inter-sample comparison [23]. Using the default settings and inputs, ESTIMATE and EPIC output the proportion of both immune and stromal compartments, CIBERSORT and quanTIseq output only the proportions of immune compartment while ISOpure output the estimation of cancer and non-cancer compartments as well as the cancer and non-cancer expression profile per sample (see the “Methods” section for details and Additional file 2: Table S1 for inputs and outputs of these methods). To evaluate their performance in estimating the cancer cell proportion, we compared their results with tumor purity estimation derived from orthogonal information, i.e., copy number (ABSOLUTE [36] [37]). As shown in Fig. 2a, the top 3 methods, DeClust, ESTIMATE, and CIBERSORT, performed similarly with regard to their correlations with ABSOLUTE’s estimates and performed significantly better than the other methods (*p* < 0.05 by two-sided paired *t* test). The DeClust purity estimates were ranked the best in term of median absolute deviation (MAD) (Fig. 2b). The immune cell scores output by CIBERSORT (the absolute version) do not reflect cell fractions (within the range of [0,1]), thus were not included in the comparison of MAD. Even though the ESTIMATE purity estimation was non-linearly transformed from ssGSEA scores using ABSOLUTE purity as the target [6], a larger systematic shift from ABSOLUTE was still observed for ESTIMATE as compared to DeClust (Fig. 2c). To further evaluate their performance in estimating immune and stromal cell proportion separately, we also compared the cell fraction estimates with the estimates generated from methylation data (MethylCIBERSORT [38]). DeClust consistently ranked among the top methods in this comparison (Additional file 1: Figure S5). Together, these results demonstrate that DeClust is among the best methods for estimating cell components.

**Fig. 3:**
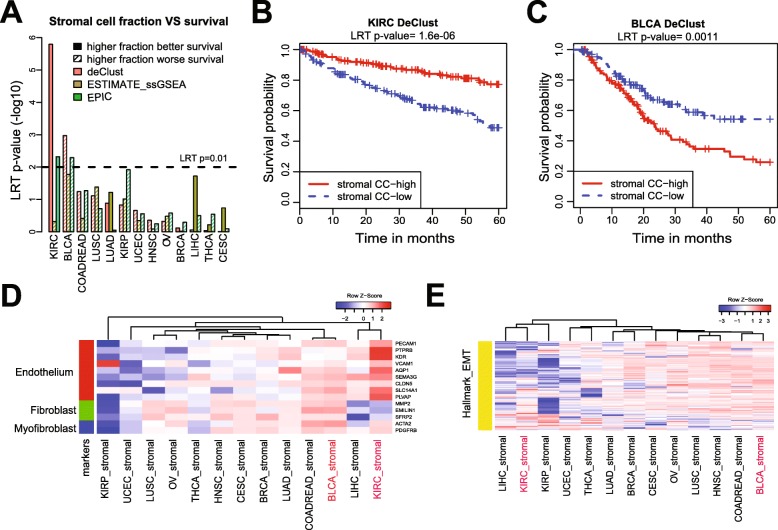
**a** Association between overall survival and stromal cell fraction estimated by different methods. Kaplan-Meier curves of patients with high/low stromal compartment fractions as defined by DeClust in the KIRC (**b**) and BLCA (**c**) TCGA datasets. Heat maps of the gene expression levels estimated by DeClust of cell type-specific markers (**d**) and EMT genes (**e**) in the stromal compartment across the 13 cancer types

Although the estimates of immune proportion showed very high correlation among different deconvolution methods and by using different genomic information, the estimates of stromal proportions were poorly correlated (Fig. 2d). This indicates that while the immune proportion can be robustly estimated using a universal immune gene signature, the stromal cell fraction may be less amenable to estimation using universal gene signatures. We thus turned further attention to the stromal compartment. Since correlation with MethylCIBERSORT could not distinguish the performance of the three methods, i.e., DeClust, ESTIMATE, and EPIC, in estimating the stromal cell proportion, we next assessed the correlations of the stromal estimates using these various approaches with clinical outcomes. Based on the stromal proportions estimated by DeClust across tumor types (Fig. 3a), a higher stromal proportion was associated with better survival in KIRC (Fig. 3b, *p* value of log rank test = 1.6e−6), whereas a higher proportion was associated with worse survival in BLCA (Fig. 3c, *p* value of log rank test = 0.0011). While the trend of association in BLCA was also supported by ESTIMATE (Additional file 1: Figure S6, *p* value of log rank test = 0.017), the association in KIRC was not detected by ESTIMATE (Additional file 1: Figure S6, *p* value of log rank test = 0.48). These trends were also less apparent using the EPIC estimates of stromal proportion. We subsequently confirmed the associations between DeClust estimates of stromal proportion and survival in independent datasets: GSE3538 [39] for KIRC and GSE32894 [40] for BLCA, respectively (Additional file 1: Figure S6, see the “Methods” section for details). Thus, using the stromal proportions estimated by DeClust, we were able to uncover survival associations that were not readily apparent with other methods.

We next assessed whether the tumor-type-specific stromal gene expression profiles estimated by DeClust could elucidate our understanding of the distinctive effects the stromal compartment proportion on survival outcomes in BLCA and KIRC. We examined the stromal gene expression profiles inferred by DeClust across the 13 tumor types of known cell-type-specific markers [25, 28] (Fig. 3d). There was an increased expression of endothelial cell markers in the KIRC’s stroma profile while fibroblast and myofibroblast cell markers demonstrated increased expression in the stromal profiles for BLCA (Fig. 3d). We also compared the pathway activity among the 13 stromal profiles (Additional file 1: Figure S7). Consistent with our prior report [41], genes in “epithelial and mesenchymal transition” (EMT) and “myogenesis” pathways were the most upregulated in the stromal compartment expression profile of BLCA compared to the other cancer types (Fig. 3e). Thus, the distinct effects of the stromal proportion on survival outcomes may, at least in part, result from the differential cellular composition and pathway activity of the stroma in BLCA and KIRC. DeClust was able to facilitate such discoveries by inferring the tumor-type-specific stromal expression profiles.

### DeClust identified cancer subtypes across 13 tumor types

**Fig. 4:**
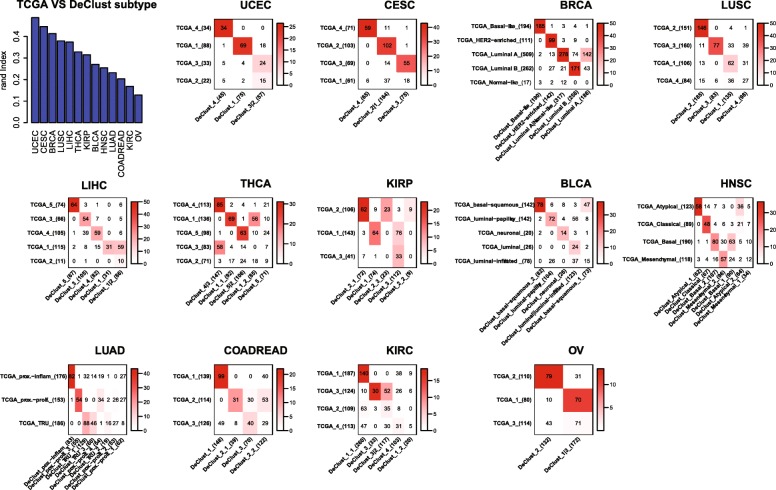
Overlap between the DeClust and TCGA subtypes. Numbers in the overlap table represent the number of samples shared by the two subtypes, and the color intensity represents the significance of the overlap

The cancer cell-intrinsic subtype outputs from DeClust were annotated according to their best overlap with the clustering results reported by TCGA (Fig. 4, Additional file 3: Table S3). The difference between TCGA and DeClust subtypes may be attributed to many factors: different clustering strategies and different numbers of subtypes as well as the effects of dissecting the immune and stromal compartments. To better illustrate the effects of deconvolution which is the main focus of this study, we applied *K*-means clustering directly to the TCGA bulk tissue expression data with the same number of subtypes chosen by DeClust. To explore alternative strategies in deriving cancer cell-intrinsic subtypes, we also applied the two-step strategy described in the simulation section. Briefly, EPIC or CIBERSORT was used to estimate the cell components, based on which the non-cancer compartment was subtracted from the bulk tissue expression. The *K*-means clustering was then applied to the residual expression profiles (i.e., cancer compartment). As ISOpure outputs not only the cell component estimation but also the estimated cancer expression profile per sample, *K*-means algorithm was applied directly to the latter. In all the above alternative strategies, the same optimal subtype number selected by DeClust was used to avoid bias due to different numbers of subtypes. We compared different subtype annotations with respect to subtype-specific DNA variations, prognosis, and variations in stromal and immune cell-intrinsic properties.

**Fig. 5:**
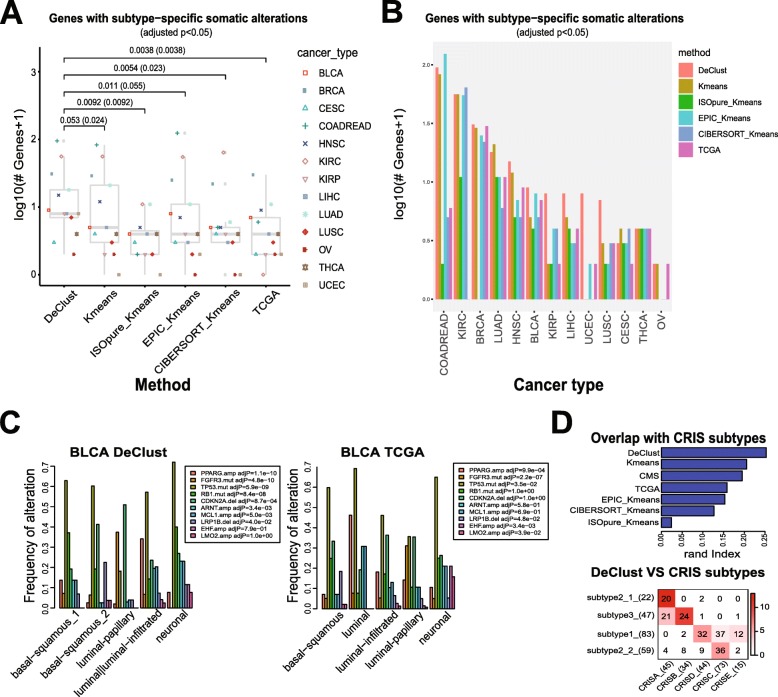
Plots of the numbers of somatically mutated, amplified, or deleted genes that were enriched in subtypes defined by different methods (adjusted *p* value of chi-squared test < 0.05) grouped by methods (**a**) or by cancer types (**b**). The *p* values above are the difference between DeClust and other methods according to the two-sided paired Wilcoxon rank-sum test based on the log-transformed counts or the original counts (in parenthesis). **c** The frequency of genetic alterations within each BLCA subtype defined by DeClust (left) or TCGA (right). Only genetic alterations that were enriched in either DeClust or TCGA subtypes in BLCA are included here (adjusted *p* value < 0.05). **d** Similarity between CRIS subtypes and subtypes derived from different methods for COADREAD (upper) and overlap between CRIS subtypes and DeClust subtypes (lower)

Cancer molecular subtypes driven by cancer cell-intrinsic properties as opposed to variations in non-cancer cell compartments are likely to be characterized by subtype-specific genomic alterations (somatic mutations or copy number alterations) [42, 43]. Better subtype classifications would lead to more enrichment for such alterations. Thus, we compared subtypes generated by different methods with respect to enrichment of subtype-specific somatic mutations or copy number alterations. DeClust subtypes were ranked the highest among all methods assessed here, suggesting that DeClust subtypes were more likely driven by cancer cell-intrinsic properties (with statistical significance *p* < 0.05 or marginal significance *p* < 0.1 in Fig. 5a, b). Some well-known subtype-specific mutations were more enriched in DeClust subtypes compared with TCGA subtypes (Additional file 4: Table S4). For example, FGFR3 mutations, one of the best characterized features of luminal-papillary bladder cancer [44], were present in 37% of the luminal-papillary subtype based on DeClust, versus 31% by TCGA (Fig. 5c). The *K*-means results based on the TCGA bulk tissue expression profiles were ranked second. The numbers of subtype-specific genomic alterations were lower compared with subtypes based on DeClust (*p* = 0.024 using the original gene counts or 0.053 using the log-transformed gene counts by two-sided paired Wilcoxon rank-sum test), but higher than based on subtypes reported by TCGA. The better performance of the *K*-means on the bulk tissue expression profiles might be partially attributed by the differences of the optimal subtype numbers identified by DeClust and TCGA. For instance, the number of subtype-specific mutations in COADREAD would be much lower for the *K*-means method if *K* = 3 (based on TCGA) was used as opposed to *K* = 4 (based on DeClust) [47 vs 82]. Although the results based on EPIC_Kmeans and CIBERSORT_Kmeans were not better than the *K*-means results overall in terms of the number of subtype-specific genomic alterations, there are noticeable exceptions, e.g., the EPIC_Kmeans result ranked the highest for COADREAD and the CIBERSORT_Kmeans result ranked the highest for KIRC (Fig. 5b).

Methylation is another common type of genomic alteration in cancer cells. However, variation in the methylation level of bulk tissue profiles could be due to both variation of cellular compositions (the foundation for MethylCIBERSORT) and cancer cell-intrinsic alterations. Indeed, DeClust subtypes showed the highest numbers of subtype-specific methylations only after methylations associated with immune or stromal cell frequencies were filtered out (Additional file 1: Figure S8).

A stronger subtype-specific association with genomic alterations is only an indirect indication that the subtypes are likely to be driven by cancer cell-intrinsic genomic alterations. A more direct assessment would be to compare them with cancer subtypes derived directly from cancer cells or patient-derived xenograft (PDX) models. A recent study [45] utilized gene expression data from PDX models to derive five cancer cell-intrinsic subtypes in colorectal cancer (CRIS). We thus compared subtypes based on DeClust and other methods with CRIS subtypes. As shown in Fig. 5d and Additional file 1: Figure S9, DeClust subtypes showed the highest overlap with CRIS subtypes among all the subtypes assessed here, including the consensus molecular subtypes (CMS) which were pooled from six independent classification systems [46].

**Fig. 6:**
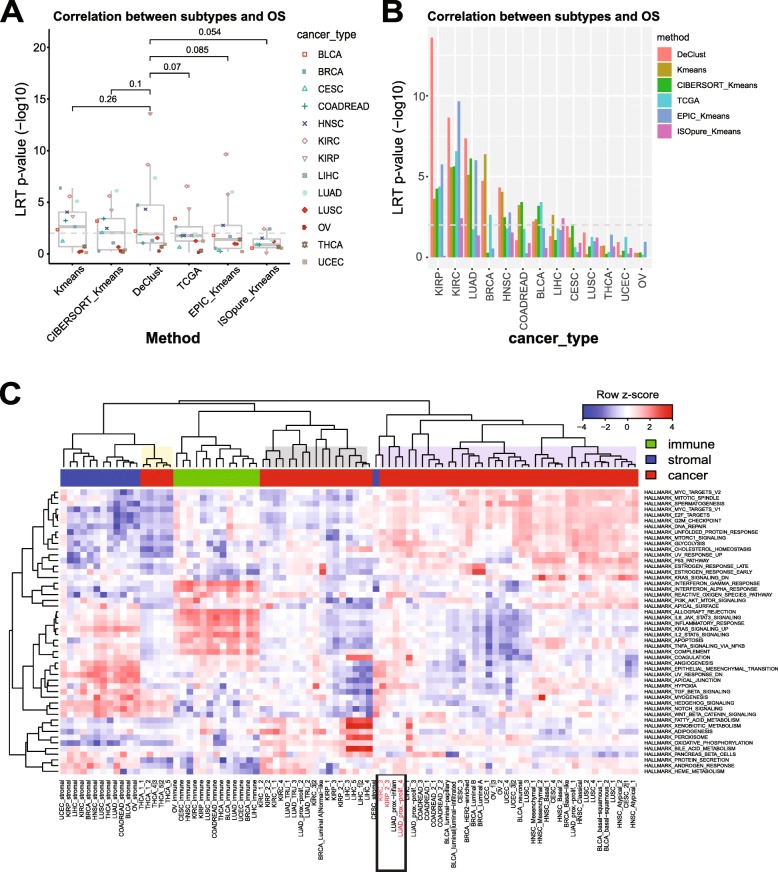
Plots of associations between overall survival outcome and subtypes defined by different methods grouped by methods (**a**) or by cancer types (**b**). The *p* values above are the difference between DeClust and other methods according to the two-sided paired *t* test. **c** Heat map of the pathway activity in the immune, stromal, and cancer compartment across 13 TCGA datasets based on the expression profiles of these compartments as estimated by DeClust. The pathway activity score was calculated by single-sample gene set enrichment analysis (ssGSEA) [24, 47] and then row-wise scaled for display. We considered 50 cancer Hallmark pathways annotated in MsigDB by Broad Institute [48]

Patients with different cancer molecular subtypes are likely to have different prognoses [42, 43]. We thus compared the association between patient prognosis and the subtypes identified by different methods. Across the 13 datasets, DeClust subtypes overall performed similar to the other approaches (Fig. 6a) but may be favored for cancer types with the strongest subtype-specific associations with survival (Fig. 6b). The top three cancer types were investigated in more details later. We also showed that DeClust subtypes were significantly less correlated with non-cancer cell-intrinsic variations, such as sample purity, compared with other subtypes (Additional file 1: Figure S10). In summary, compared to TCGA subtypes, DeClust subtypes were more likely driven by cancer cell-intrinsic genetic alterations as opposed to non-cancer cell variations, in addition to having a stronger association with survival outcomes in certain tumor types.

### Clinically relevant outlier subtypes identified by DeClust

To characterize the cancer cell subtype-specific gene expression profiles output by DeClust, we summarized the gene expression profiles at the pathway level (Fig. 6b, detailed in the “Methods” section). The cancer cell subtype-specific profiles could largely be grouped into three clusters characterized by overexpression of cell cycle pathways (purple), metabolic pathways (gray), or stromal-related genes (yellow). Cancer subtypes of the same tumor type generally clustered together reflecting tissue of origin-related specificity. For example, the Wnt signaling pathway was upregulated in COARDREAD cancer subtypes [49], the estrogen response pathway was upregulated in the majority of BRCA subtypes [50] (with the exception of the basal-like subtype), and the fatty acid metabolism pathway was upregulated in LIHC subtypes. Most cancer cell subtype profiles were clustered together based on higher activity in cell cycle-related genes, with the exception of thyroid cancer, which was separated from other cancer subtype profiles given it is a slow growing cancer with low cell cycle activity.

**Fig. 7:**
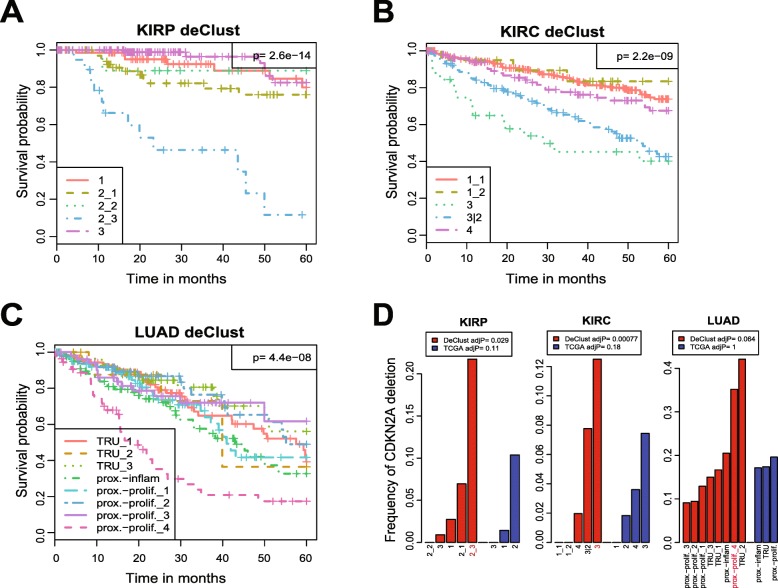
Kaplan-Meier curves of DeClust subtypes for the KIRP (**a**), KIRC (**b**), and LUAD (**c**) TCGA datasets. **d** Frequency of CDKN2A deletions in each subtype of KIRP, KIRC, and LUAD as defined by DeClust and TCGA

While most cancer subtypes of the same tumor type clustered together, a group of “outlier” subtypes was observed, consisting of different tumor types with shared underlying pathway activities. For example, KIRC_3, KIRP_2_3, and LUAD_proxi-prolif_4 were clustered together (Fig. 6b). Strikingly, these three subtypes were also associated with significantly worse survival outcomes compared with the other subtypes of the corresponding tumor types (Fig. 7a–c). Interestingly, all were enriched for *CDKN2A* deletions (Fig. 7d). This is consistent with the previous discovery that samples with the same tumor suppressor gene inactivation in different cancer types tended to cluster together [51]. The associations of the worse survival and the outlier DeClust subtypes of KIRP, KIRC, and LUAD were replicated in independent datasets GSE2748 [52], GSE3538 [39], and GSE31210 [53], respectively (Additional file 1: Figure S11). These findings highlight the potential of DeClust to identify heretofore unrecognized intrinsic cancer subtypes of potential clinical relevance.

### Comparing stromal expression profiles inferred by DeClust and single-cell RNAseq data

**Fig. 8:**
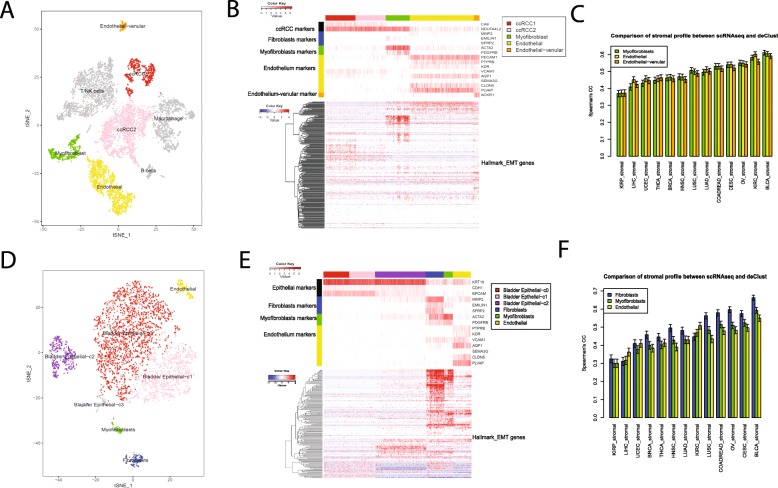
Comparison of scRNAseq data and DeClust tumor-type-specific stromal profiles. Clustering results of scRNAseq data for ccRCC samples (**a**) and BLCA samples (**d**). Cells are colored according to cell clusters. Expression levels (heat map) of cell type-specific markers (top) and EMT genes (bottom) in each cell of the RNAseq data for ccRCC (**b**) or BLCA (**e**). Marker gene expression levels are represented as the absolute expression value, log2(read count+ 1), while expression levels for EMT genes are represented as the row-wise scaled relative expression value. For display purpose, cells in ccRCC2 were downsampled to the same size of ccRCC1. Cells in Epithelia-c0 and Epitheila-c1 were downsampled so that the combined cell number in the two clusters equals to that in Epithelial-c2. Correlation between stromal profiles estimated by DeClust and stromal profiles calculated by mean expression profile of each stromal cell cluster in scRNAseq data of ccRCC (**c**) or BLCA (**f**). Error bars indicate the 95% confidence interval

DeClust estimated the immune and stromal expression profiles in a cancer-type-specific manner. We first compared these imputed expression profiles with known reference expression profiles of immune and stromal cells that are agnostic to the cancer types to demonstrate its general effectiveness (see Additional file 1: Figure S12 and Additional file 2: Supplementary Methods and Results). To further evaluate the tumor-type-specific stromal profiles inferred by DeClust, we compared them with single-cell RNA sequencing (scRNAseq) data. In particular, we looked into KIRC and BLCA since their stromal profiles exhibited disparate prognostic effects (Fig. 3a), and suggested different compositions of stromal cells (Fig. 3e). We collected scRNAseq data generated on clear cell renal cell carcinoma (ccRCC, referred hereafter as KIRC) from a previous study [25]. There were three clusters of cells from the stromal compartment in the KIRC scRNAseq dataset: endothelial cells, myofibroblasts, and venular endothelial cells (Fig. 8a, b). The largest fraction (68%) for the stromal compartment was endothelial cells, and no classical fibroblast cells were identified in this dataset. The KIRC stromal profile inferred by DeClust correlated best with the average endothelial cell profile based on the scRNAseq dataset compared with other stromal cell profiles (Fig. 8c).

Noting that the KIRP’s DeClust_stromal profile was poorly correlated with all three types of the stroma cell profiles (Fig. 8c, f), we collected and analyzed scRNAseq data of papillary renal cell carcinoma (pRCC) [25] in a similar way to that of scRCC. There was no clear cluster of stromal cells in pRCC’s microenvironment (Additional file 1: Figure S13), which may partially explain the low correlations between KIRP’s DeClust_stromal profile and stroma cell profiles (Fig. 8c).

As there are no publicly available BLCA scRNAseq datasets, we performed scRNAseq profiling on two fresh muscle-invasive bladder cancer specimens in-house (detailed in the “Methods” section deposited as GSE130001 [21]). Cells in the stromal compartment of the BLCA scRNAseq dataset were clustered into fibroblasts (40%), myofibroblasts (20%), and endothelial cells (40%) (Fig. 8d, e). The DeClust inferred BLCA stromal expression profile correlated best with the fibroblast cell profile in the BLCA scRNAseq dataset (Fig. 8f).

Thus, the scRNAseq data indicates different stromal cellular compositions in different cancer types. For example, there was a relatively higher proportion of endothelial cells in KIRC and a relatively higher proportion of fibroblasts in BLCA, consistent with the observations in Fig. 3e. In addition, the EMT signal in BLCA stromal compartment (Fig. 3f) was at least partially driven by fibroblasts (Fig. 8e).

### Comparing DeClust cancer profiles and single-cell RNAseq data

**Fig. 9:**
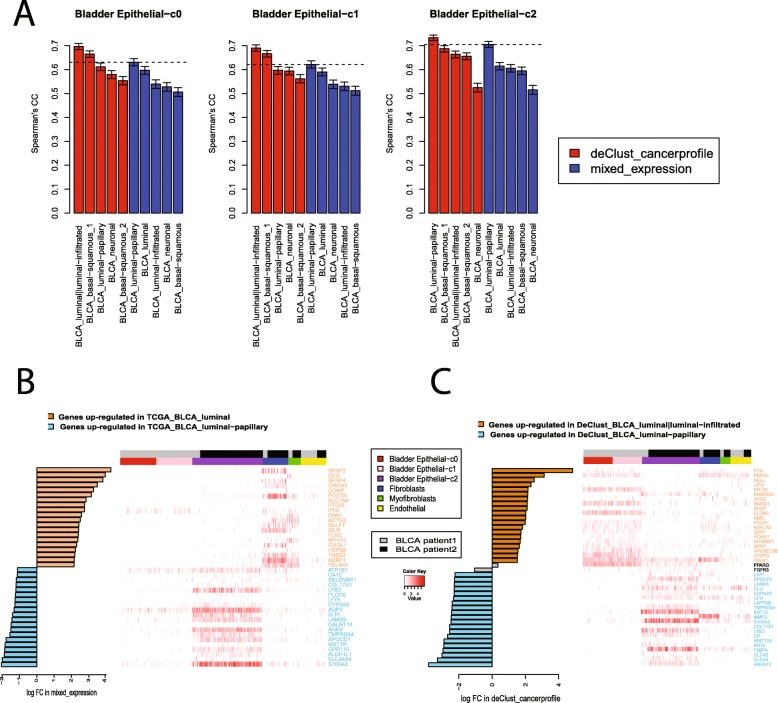
Comparison of BLCA scRNAseq data and BLCA subtypes. **a** The correlation between mean expression profile of each epithelial cell cluster and subtype-specific cancer profiles estimated by DeClust or by TCGA. Error bars indicate the 95% confidence interval. **b** Bar plot of the fold change of the top 20 up/downregulated genes between two BLCA luminal subtypes defined by TCGA (left) and the heat map of their expression in our scRNAseq dataset (right). The fold change was calculated based on mixed expression values. The absolute expression values (log-transformed) are showed in the heat map. **c** Similar to **b** except the top 20 genes and their fold change was derived by comparing the two luminal subtypes defined by DeClust. The fold change was calculated by comparing the subtype-specific profiles estimated by DeClust

Our cancer subtype comparison between DeClust and TCGA described above examined associations with genomic alterations and prognosis. To more directly compare the molecular profiles inferred by DeClust for each subtype to known molecular states of these subtypes, we compared profiles for cancer cell clusters derived from available scRNAseq datasets (i.e., BLCA, KIRC, and KIRP) with the cancer subtype profiles inferred by DeClust, in addition to those derived from the bulk tissue cancer profiles based on TCGA subtypes (Fig. 9a and Additional file 1: Figure S14). As expected, the scRNAseq cancer profiles generally correlated better with DeClust cancer profiles than to the TCGA bulk tissue profiles, given the latter are confounded by non-cancer cell compartments. For some cancer cell clusters in the scRNAseq datasets, the best matched cancer subtypes were consistent. For example, bladder cancer epithelial cell cluster c2 (shown in Fig. 8d) was the most similar to the luminal-papillary subtype assessed by both DeClust and TCGA profiles (Fig. 9a). However, for other cancer cell clusters, discrepancies were observed. For instance, the bladder cancer epithelial cell clusters c0 and c1 were best matched with the luminal|lumina-infiltrated subtype based on the DeClust profiles, but were matched with the luminal-papillary subtype based on the TCGA profiles (Fig. 9a).

To understand this discrepancy, we utilized the scRNAseq data to dissect which cell types contributed most to the differences between the different subtypes. We compared the gene signatures of the TCGA luminal-papillary and TCGA luminal subtypes, identified the top 20 most upregulated genes in each subtype, and then examined the expression of these genes in our bladder cancer scRNAseq data (Fig. 9b). The top 20 genes upregulated in the TCGA luminal subtype were most highly expressed in stromal cells, suggesting signals in stroma cells instead of bladder epithelial cells contributed to differences in the TCGA subtypes. On the other hand, the top 20 genes upregulated in the TCGA luminal-papillary subtype were most highly expressed in the Epithelial-c2 cluster, but not in Epithelial-c0 and Epithelial-c1 despite all three clusters being annotated as TCGA_luminal-papillary. We performed a similar analysis comparing the gene expression profiles of DeClust_luminal|luminal-infiltrated and DeClust_luminal-papillary subtypes and identified the top 20 most upregulated genes in each subtype. The top 20 genes upregulated in the DeClust_luminal|luminal-infiltrated subtype were highly expressed in cancer cells in the Epithelial-c0 and Epithelial-c1 clusters, and the top 20 genes upregulated in DeClust_luminal-papillary were highly expressed in cancer cells in the Epithelial-c2 cluster (Fig. 9c). Similar findings were observed using pathway analysis (Additional file 1: Figure S15). Together, these findings suggest that non-cancer cells in bulk tissue transcriptomes contribute to conventional BLCA subtypes (Additional file 1: Figure S16) and that DeClust subtypes may better reflect cancer cell-intrinsic differences.

## Discussion

We developed a reference profile-free deconvolution algorithm, DeClust, that estimates both cell fractions and reference expression profiles for different compartments of the tumor, as well as generating cancer cell-intrinsic subtyping. The unique feature of DeClust is that it directly models inter-tumor heterogeneity and integrates cancer subtypes into deconvolution models to improve the accuracy of both cell compartment deconvolution and subtyping. The advantages of such a strategy were demonstrated by comparing with various deconvolution methods and alternative strategies for cancer subtyping in both simulated and TCGA datasets. In simulated datasets, DeClust achieved comparable accuracy with the reference-based method (CIBERSORT) in estimating cell compartment frequency, but outperformed CIBERSORT_Kmeans with respect to inferring cancer cell-intrinsic subtypes. Further, DeClust was demonstrated to be able to reliably estimate compartment-specific reference profiles. We note, however, that given the simulated datasets are simplified representations of the inter- and intra-tumor heterogeneity in real data, the accuracy achieved here is likely overestimated for both DeClust and CIBERSORT.

To test performance on real data, we applied DeClust and a few other alternative methods to 13 TCGA datasets. DeClust estimated tumor purity with accuracy comparable to the best methods assessed in this study, with the particular benefit of having the smallest MAD from orthogonal measurements. Because different methods vary with respect to their definition of tumor purity, and because there is presently no gold standard, definitive comparisons were limited. Nevertheless, DeClust demonstrated a number of advantages. First, DeClust uncovered a significant association between the stromal cell proportion and patient survival outcomes in KIRC that was not apparent using other methods. Second, stromal profiles estimated by DeClust for each of the 13 TCGA datasets helped shed light on the heterogeneity of stromal compartments across tumor types. For example, KIRC and BLCA stromal profiles indicated distinct stromal cell compositions and pathway activities, which we corroborated with scRNAseq data. However, further studies are needed to demonstrate whether such differences in cellular composition are causally linked to their disparate effects on prognosis. Finally, we demonstrated direct generation of cancer cell-intrinsic subtypes, a unique attribute of DeClust. Compared to the subtypes inferred by TCGA or other methods assessed in the paper, the DeClust subtypes better reflected the contribution of cancer cells, as evidenced by an increased association with somatic alterations and decreased association with tumor purity. Since identifying subtype-specific genetic alterations is an important strategy for identification of potential driver genes and associated therapeutic targets [42, 43], DeClust could be a useful tool for such purposes, and helping to uncover genetic associations that are otherwise obscured by the non-cancer cell compartments.

Whether cancer cell-intrinsic subtypes, subtypes based on other cellular components of the microenvironment, or their interactions are more clinically relevant likely depends at least in part on the specific type of cancer. DeClust identified poor prognosis subtypes in KIRC, KIRP, and LUAD, which were enriched for *CDKN2A* deletions, highlighting that for these cancer types, cancer cell-intrinsic molecular programs are key drivers of prognosis. On the other hand, the BLCA TCGA subtypes, based on the contribution of various cellular elements, were better associated with survival than the DeClust subtypes (Fig. 6b), indicating the stromal and immune compartments of BLCA are key contributors to prognosis (Fig. 3a, c). Indeed, key signals contributing to BLCA TCGA subtyping were from fibroblasts (Fig. 9b) while key signals to DeClust subtyping were from cancer cells (Fig. 9c) based on our scRNAseq data.

It is worth noting that cancer cell-intrinsic subtypes and immune subtypes were independent (Additional file 1: Figure S17) and cancer cell-intrinsic subtypes were generally more closely linked with patient survival (Additional file 1: Figure S18) (detailed in Additional file 2: Supplementary Methods and Results). However, the impact of heterogeneity in the TME on survival was at times only apparent within the context of specific DeClust subtypes highlighting the complex interplay between the cancer cellular compartment with other cellular components of the TME (Additional file 1: Figure S18, S19 and S20). Thus, combining cancer cell-intrinsic subtyping with subtyping based on TME might be the optimal strategy and our model can be further extended to address such an approach.

The pre-computed DeClust subtypes and compartment-specific gene expression profiles for each of the 13 TCGA datasets (accessible through our DeClust R package) provide a useful resource for many future studies. For example, they can serve as reference profiles in the deconvolution of bulk tissue transcriptomic data. The DeClust compartment-specific expression profiles are tumor-type-specific, distinguishing them from the existing reference profiles or fixed sets of marker genes sets that are agnostic to the tumor type. The cancer subtype-specific profiles can also be used to annotate cancer cell clusters using scRNAseq data since the subtype profiles generated from bulk tissue transcriptomic data represent signals from mixed cellular composition.

There are potential limitations to DeClust. The strategy of computational dissection of tumor gene expression data into three compartments by DeClust and other methods [15, 31, 54] still underestimates the complexity of cellular heterogeneity comprising tumors and TMEs. The resolution of deconvolution could be improved by dissection into more detailed components in the future. The DeClust algorithm is potentially generalizable to resolving bulk tissue profiles across any number of compartments given more profiling data and computation power available. The current DeClust algorithm only facilitates subtyping of tumors based on the cancer cell-intrinsic gene expression profiles. However, different subtypes of non-cancer compartments have also been described [29]. Allowing simultaneous subtyping of both cancer and non-cancer cellular compartments may facilitate improved prognostic stratification and identify cross talk between cellular compartments critical to tumor pathogenesis. The same tumor could contain cancer cells representative of different subtypes as identified in our scRNAseq data. Thus, the flexibility of DeClust could be further improved by allowing a mixture of different cancer cell-intrinsic subtypes in the same specimen. The expression profiles estimated by DeClust are reference expression profiles at the cohort level and deconvolution could be carried further at the individual patient level. Our comparison of DeClust with other deconvolution methods was limited in that we used the default parameters and input signature matrix of the original algorithms, which might be less ideal for analyzing TCGA datasets or addressing the problem of cancer cell-intrinsic subtyping. Finally, we restricted the current study to 13 solid tumor datasets in TCGA where the tumor consists of the three main compartments, i.e., cancer cells, immune cells, and stromal cells (mainly fibroblasts). Not every solid tumor dataset fits this definition (Additional file 2: Supplementary Methods and Results), and further efforts are needed to adapt DeClust to address these tumor types.

## Conclusions

We developed a reference profile-free deconvolution method to infer cancer cell-intrinsic subtypes and tumor-type-specific stromal profiles. The cancer-intrinsic subtyping generated by DeClust together with the deconvolution results may lead to mechanistic and clinical insights across multiple tumor types.

## Supplementary information

**Additional file 1 : Figure S1.** A schematic illustration of DeClust algorithm. **Figure S2** Flowchart of the study. **Figure S3.** Simulation results when gene expression was simulated under negative binomial distribution. Details can be found in legend of Fig. 1. **Figure S4**. Simulation results when gene expression was simulated under negative binomial distribution. Details can be found in legend of Fig. 1. **Figure S5.** Comparing cell fraction estimations by different gene expression deconvolution methods with the ones based on MethyCIBERSORT (treated as “ground truth”) for immune compartment (AB) and stromal compartment (CD) using Spearman’s correlation coefficients (AC) or Median Absolute Deviation (BD). The *p*-values above are the difference between DeClust and other methods according the two-sided paired t-test. **Figure S6.** Kaplan-Meier curves of patients with high/low stromal compartment fractions as defined by DeClust (left) and ESTIMATE (right) in TCGA dataset (top) and non-TCGA dataset (bottom) for KIRC (A) and BLCA (B). **Figure S7.** Pathway analysis of stromal proles across 13 TCGA datasets using Canonical pathways (left) or hallmark_cancer pathways (right) from MsigDB. The color indicates the significance of the up/down-regulation of that pathway in that stromal prole as compared to other stromal proles (−log10 (p-value of Wilcoxon rank-sum test), see Method). Red color represents up-regulation and blue color represents down-regulation. **Figure S8**. Number of genes with subtype-specific methylation before (A) and after (B) methylation correlated with immune or stromal cell frequencies were removed. **Figure S9.** Overlap between CRIS subtypes and subtypes defined by different methods. **Figure S10.** Plots of the correlation between tumor purity (Consensus Purity Estimation*) and subtypes defined by different methods, grouped by methods (A) or by cancer types (B). **Figure S11.** Kaplan-Meier curves of DeClust subtypes (left) and TCGA subtypes (right) for TCGA dataset (top) and non-TCGA dataset (bottom) for KIRP (A), KIRC (B) and LUAD (C). **Figure S12**. (A) Spearman’s correlation coefficient between immune and stromal proles estimated by DeClust (y-axis) and reference proles used by EPIC (x-axis). (B) Exemplary scatter plot of reference expression prole versus immune and stromal prole estimated by Declust using TCGA BLCA dataset.(C) Correlation between different reference proles(x-axis) and bulk tissue expression proles (gray), immune proles (red) and stromal proles (blue) estimated by DeClust using TCGA BLCA dataset. (D) Cell type-specific markers identified in EPIC reference proles and their expression in immune and stromal proles estimated by DeClust. **Figure S13.** t-SNE plot of scRNAseq data for pRCC samples. Cell-type specific markers used to annotate the cell clusters were shown below. **Figure S14.** Correlation between mean expression prole of each epithelial cell cluster and subtype-specific cancer proles estimated by DeClust or by TCGA for ccRCC scRNAseq data (A) and pRCC scRNAseq data(B). Error bars indicated 95% confidence intervals. **Figure S15.** Comparison of inferred cancer proles and scRNAseq data. **Figure S16.** Violin plot of estimated immune (left) and stromal (fraction) fraction in each subtype of BLCA. **Figure S17.** Proportions of different immune subtypes within each subtypes defined by DeClust. **Figure S18.** Comparison of immune subtypes and subtypes defined by DeClust in association with overall survival. Plots are grouped by methods (A) and by cancer types(B). **Figure S19.** (A) The association of overall survival and immune subtypes within each cancer cell-intrinsic subtype defined by Declust.(B) Kaplan-Meier curves of immune subtypes within Atypical_1 subtype of HNSC. **Figure S20.** (A) The association of overall survival and immune/stromal cell fraction within each subtype defined by Declust. The color in the heatmap represents the –log10(p-value) of the association (log rank test). Red means the higher cell fraction corresponds to worse survival, and blue means the opposite. Only cancer subtypes and cell fractions with at least one significant association (*p* < 0.05) are shown here. The cell fractions were estimated by CIBERSORT, EPIC or DeClust, and both the absolute fraction (Ab) and relative fraction (Re) were assessed.(B) Kaplan-Meier curves of high and low CAF fraction within BLCA_luminal-papillary subtype. (C) Kaplan-Meier curves of high and low resting NK cells within BLCA_basal-squamous_1 subtype. **Figure S21.** BICs calculated according to the DeClust model at different subtype number for each TCGA dataset. The red dotted line indicates the number of subtypes select for further analyses. **Figure S**22 PCA plot of cancer, immune, and stroma expression profiles estimated by DeClust.

**Additional file 2 **Supplementary Methods and Results, **Table S1.** Inputs and outputs of different methods in estimation of cell composition in bulk tumor tissues. **Table S2.** mapping each immune and stromal profile onto the best matching cell in human HPCA.

**Additional file 3 Table S3**. Subtype annotation of samples in 13 TCGA datasets by DeClust or TCGA.

**Additional file 4 Table S4**. Associations between somatic mutations or copy number alterations and subtypes by DeClust or TCGA.

## Data Availability

TCGA [16] datasets are available in Broad Institute Firehose (https://gdac.broadinstitute.org/) (2016_01_28). CCLE [19] gene expression data are available in Broad Institute (https://broadinstitute.org/ccle/). The non-TCGA tumor expression datasets used in the study are available in GEO [18] database: GSE2748 [49], GSE3538 [36], and GSE31210 [50], GSE37614 [21], GSE3538 [36], GSE32894 [37]. The scRNAseq data of two BLCA tumors are deposited into GEO database (GSE130001 [21]). The methods of EPIC, quanTIseq, and the absolute version of CIBERSORT are available in R package *immunedeconv* (V2.0.0) [20] (https://github.com/icbi-lab/immunedeconv). The method of ISOpure [14] is available in R package *ISOpureR* (V1.1.3) (https://cran.r-project.org/web/packages/ISOpureR/index.html) [14]. CIBERSORT [8] method is available at https://cibersort.stanford.edu/. The DeClust methods and source codes are available in the R package DeClust (https://github.com/integrativenetworkbiology).

## Abbreviations

BLCA: Urothelial bladder carcinoma
CCLE: Cancer cell line encyclopedia
ccRCC: Clear cell renal cell carcinoma
EMT: Epithelial and mesenchymal transition
KIRC: Kidney renal clear cell carcinoma
KIRP: Kidney renal papillary cell carcinoma
LUAD: Lung adenocarcinoma
MAD: Mean absolute deviation
MSE: Mean square error
PDX: Patient-derived xenograft
pRCC: Papillary renal cell carcinoma
scRNAseq: Single-cell RNAseq
ssGSEA: Single-sample gene set enrichment analysis
