# Supplementary material for: A reference profile-free deconvolution method to infer cancer cell-intrinsic subtypes and tumor-type-specific stromal profiles
Source: Genome Med. 2020 Feb 28;12:24. doi: 10.1186/s13073-020-0720-0 (PMC7049190; doi:10.1186/s13073-020-0720-0)
Supplement: Supplementary file 1 — Additional file 1 : Figure S1. A schematic illustration of DeClust algorithm. Figure S2 Flowchart of the study. Figure S3. Simulation results when gene expression was simulated under negative binomial distribution. Details can be found in legend of Fig. 1. Figure S4. Simulation results when gene expression was simulated under negative binomial distribution. Details can be found in legend of Fig. 1. Figure S5. Comparing cell fraction estimations by different gene expression deconvolution methods with the ones based on MethyCIBERSORT (treated as “ground truth”) for immune compartment (AB) and stromal compartment (CD) using Spearman’s correlation coefficients (AC) or Median Absolute Deviation (BD). The p-values above are the difference between DeClust and other methods according the two-sided paired t-test. Figure S6. Kaplan-Meier curves of patients with high/low stromal compartment fractions as defined by DeClust (left) and ESTIMATE (right) in TCGA dataset (top) and non-TCGA dataset (bottom) for KIRC (A) and BLCA (B). Figure S7. Pathway analysis of stromal proles across 13 TCGA datasets using Canonical pathways (left) or hallmark_cancer pathways (right) from MsigDB. The color indicates the significance of the up/down-regulation of that pathway in that stromal prole as compared to other stromal proles (−log10 (p-value of Wilcoxon rank-sum test), see Method). Red color represents up-regulation and blue color represents down-regulation. Figure S8. Number of genes with subtype-specific methylation before (A) and after (B) methylation correlated with immune or stromal cell frequencies were removed. Figure S9. Overlap between CRIS subtypes and subtypes defined by different methods. Figure S10. Plots of the correlation between tumor purity (Consensus Purity Estimation*) and subtypes defined by different methods, grouped by methods (A) or by cancer types (B). Figure S11. Kaplan-Meier curves of DeClust subtypes (left) and TCGA subtypes (right) for TCGA dataset (top) and non-TC [file 13073_2020_720_MOESM1_ESM.pdf]

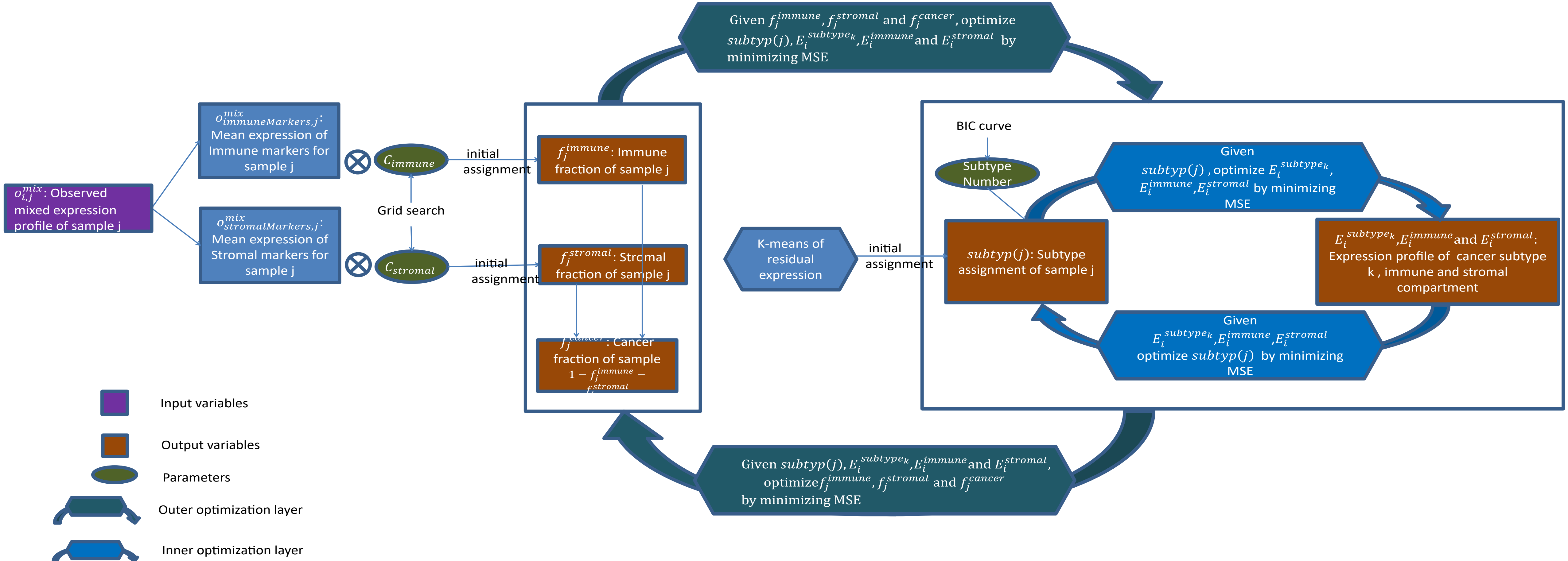

$$\mathbf{MSE} = \sum_{i,j}\left(o_{i,j}^{mix} - \log(\,exp^{E_i^{subtype_k}} \times f_j^{cancer} + exp^{E_i^{immune}} \times f_j^{immune} + exp^{E_i^{stromal}} \times f_j^{stromal})\right)^2$$

Fig. S1 A schematic illustration of DeClust algorithm.

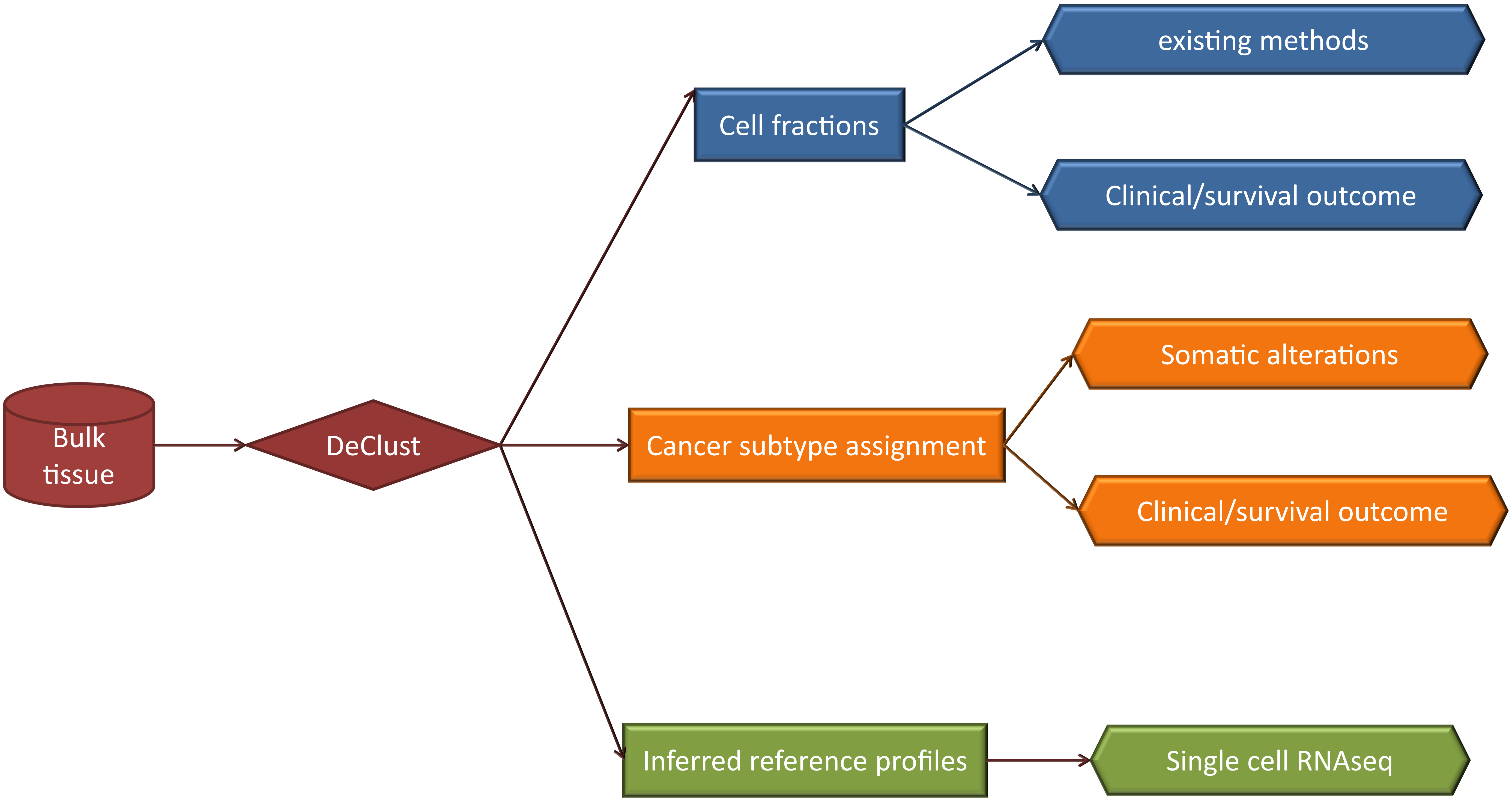

Fig. S2 Flowchart of the study.

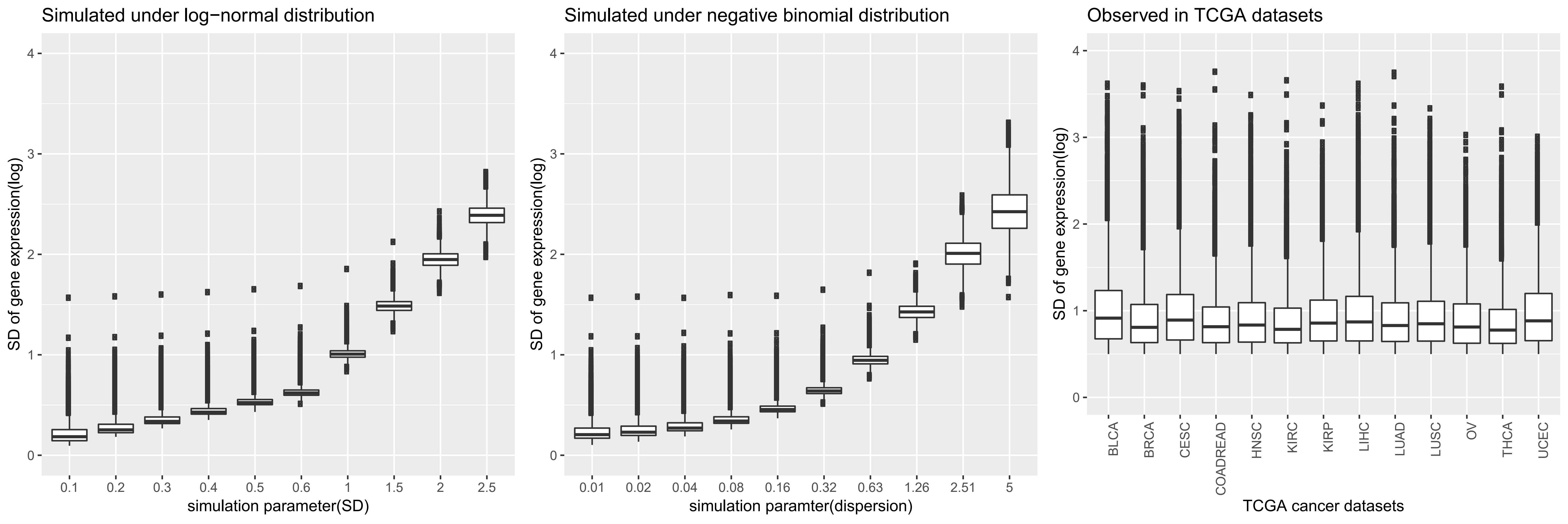

Fig. S3 Simulation results when gene expression was simulated under negative binomial distribution. Details can be found in legend of Figure 1.

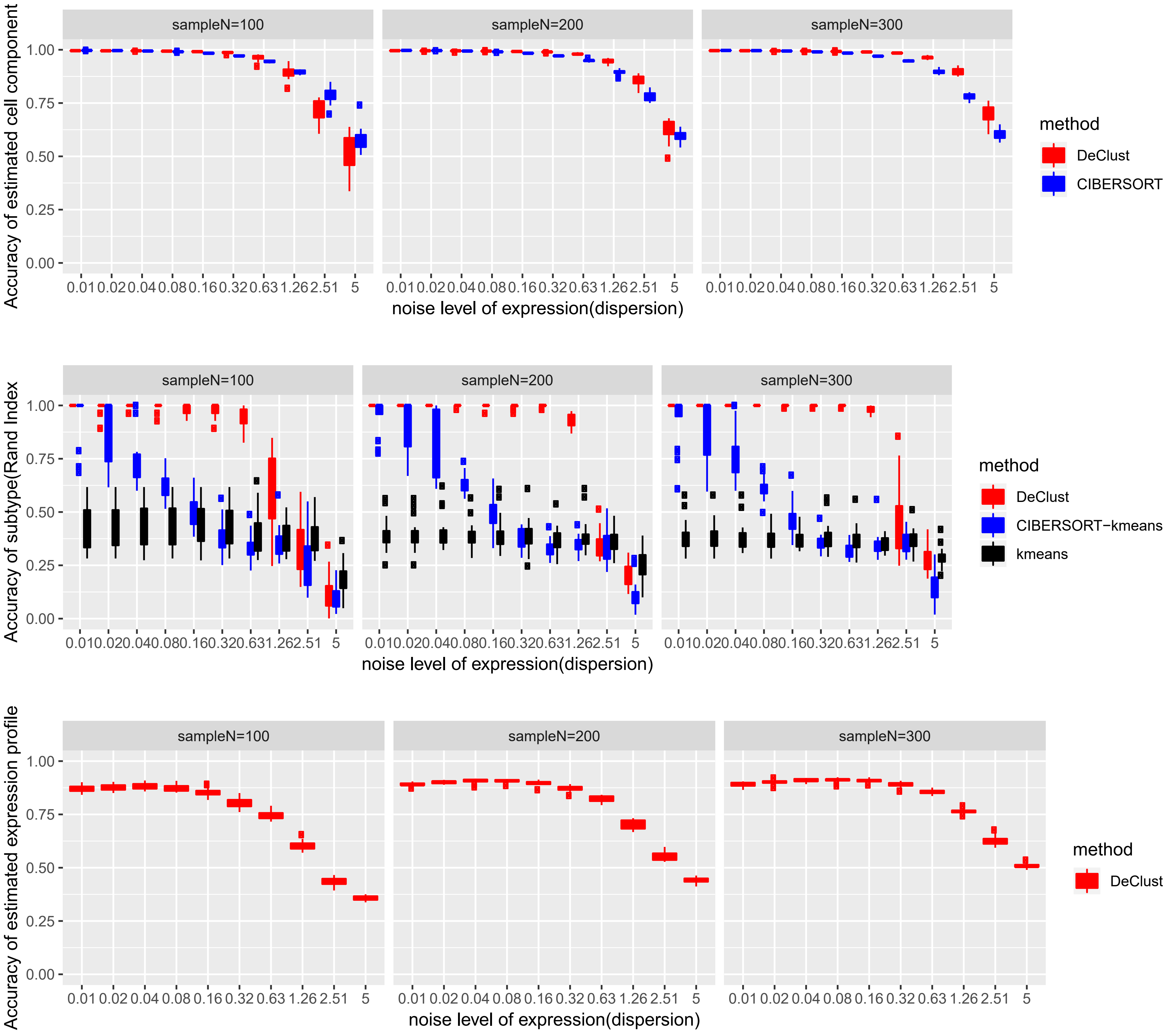

Fig. S4 Simulation results when gene expression was simulated under negative binomial distribution. Details can be found in legend of Figure 1.

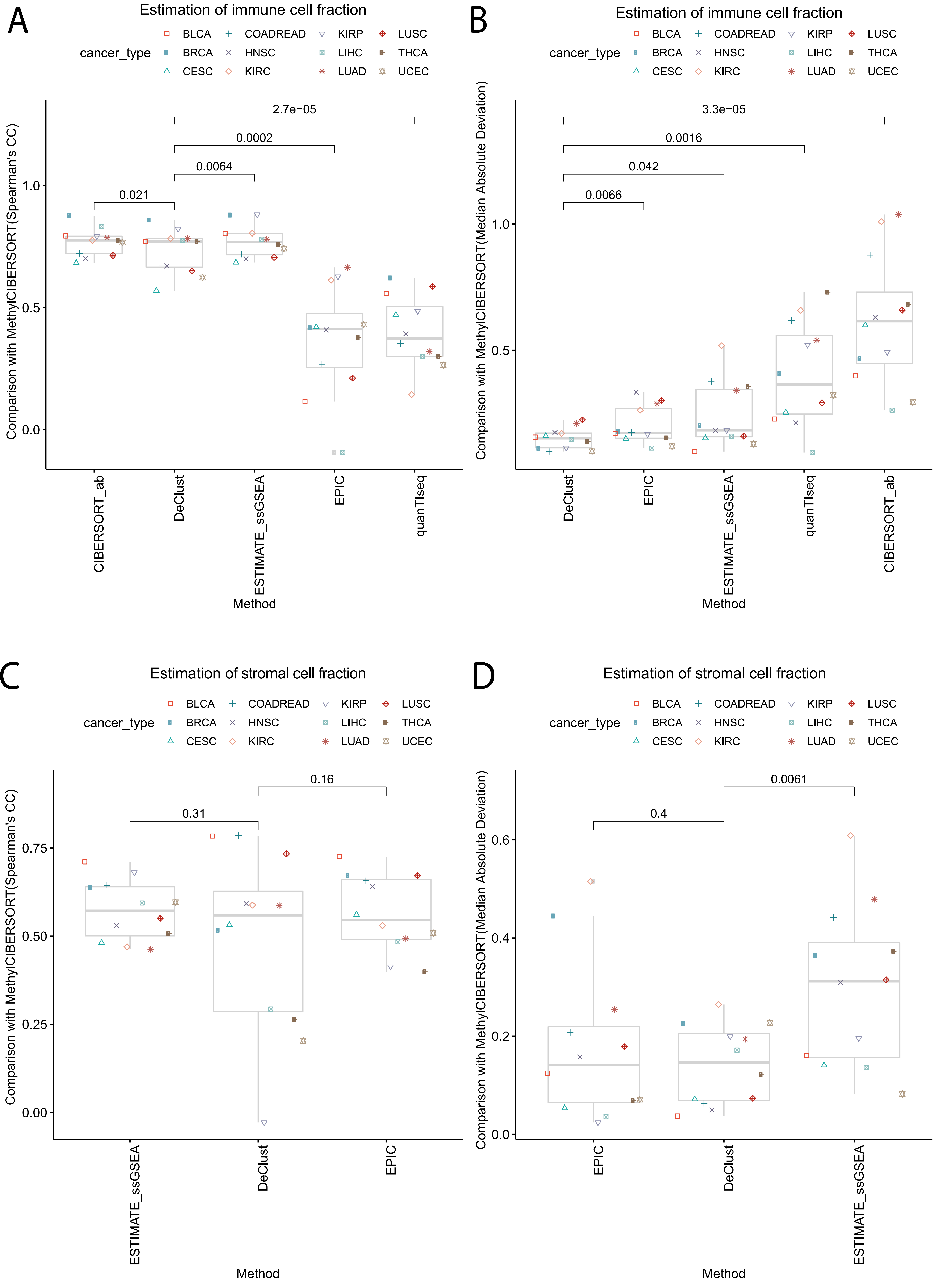

Fig. S5 Comparing cell fraction estimations by different gene expression deconvolution methods with the ones based on MethyCIBERSORT (treated as "ground truth") for immune compartment (AB) and stromal compartment (CD) using Spearman's correlation coefficients (AC) or Median Absolute Deviation (BD). The p-values above are the difference between DeClust and other methods according the two-sided paired t-test.

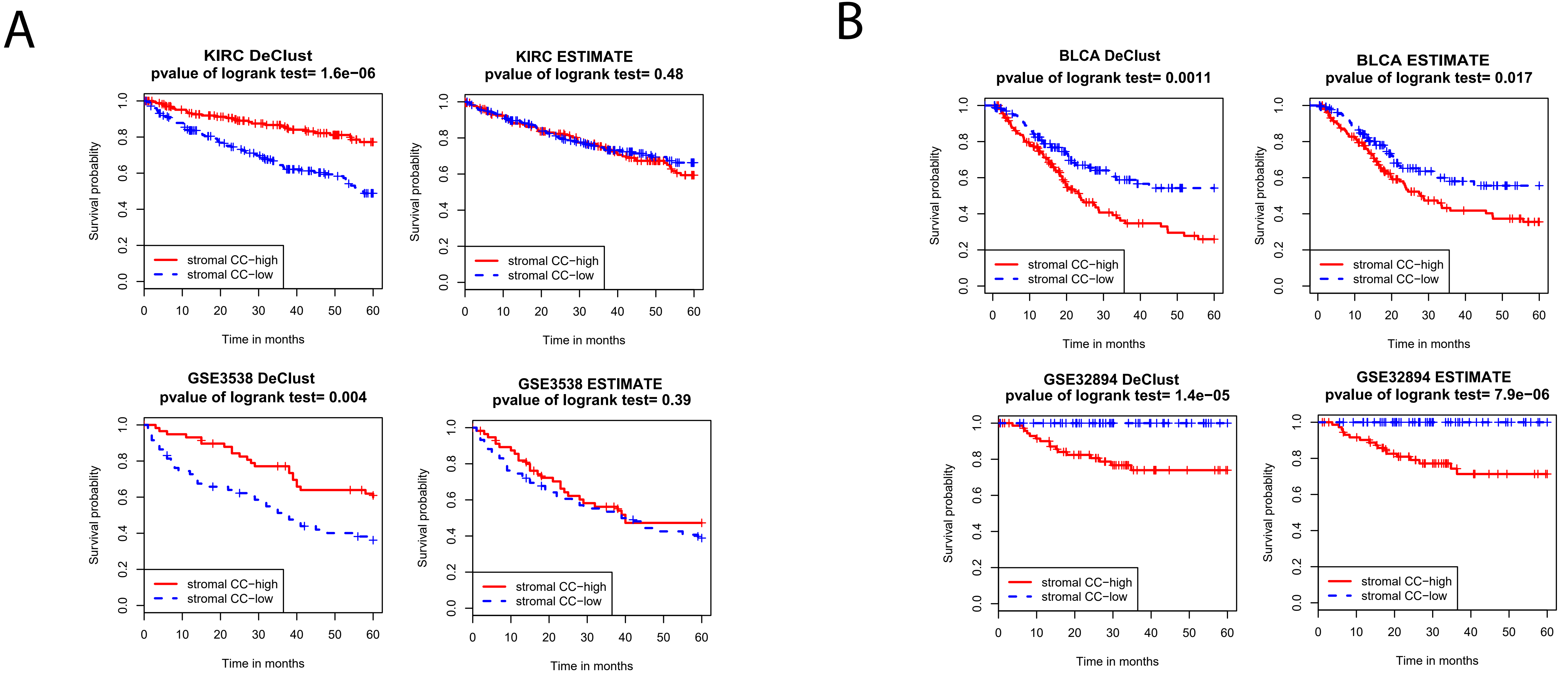

Fig. S6 Kaplan-Meier curves of patients with high/low stromal compartment fractions as defined by DeClust (left) and ESTIMATE (right) in TCGA dataset (top) and non-TCGA dataset (bottom) for KIRC (A) and BLCA (B).

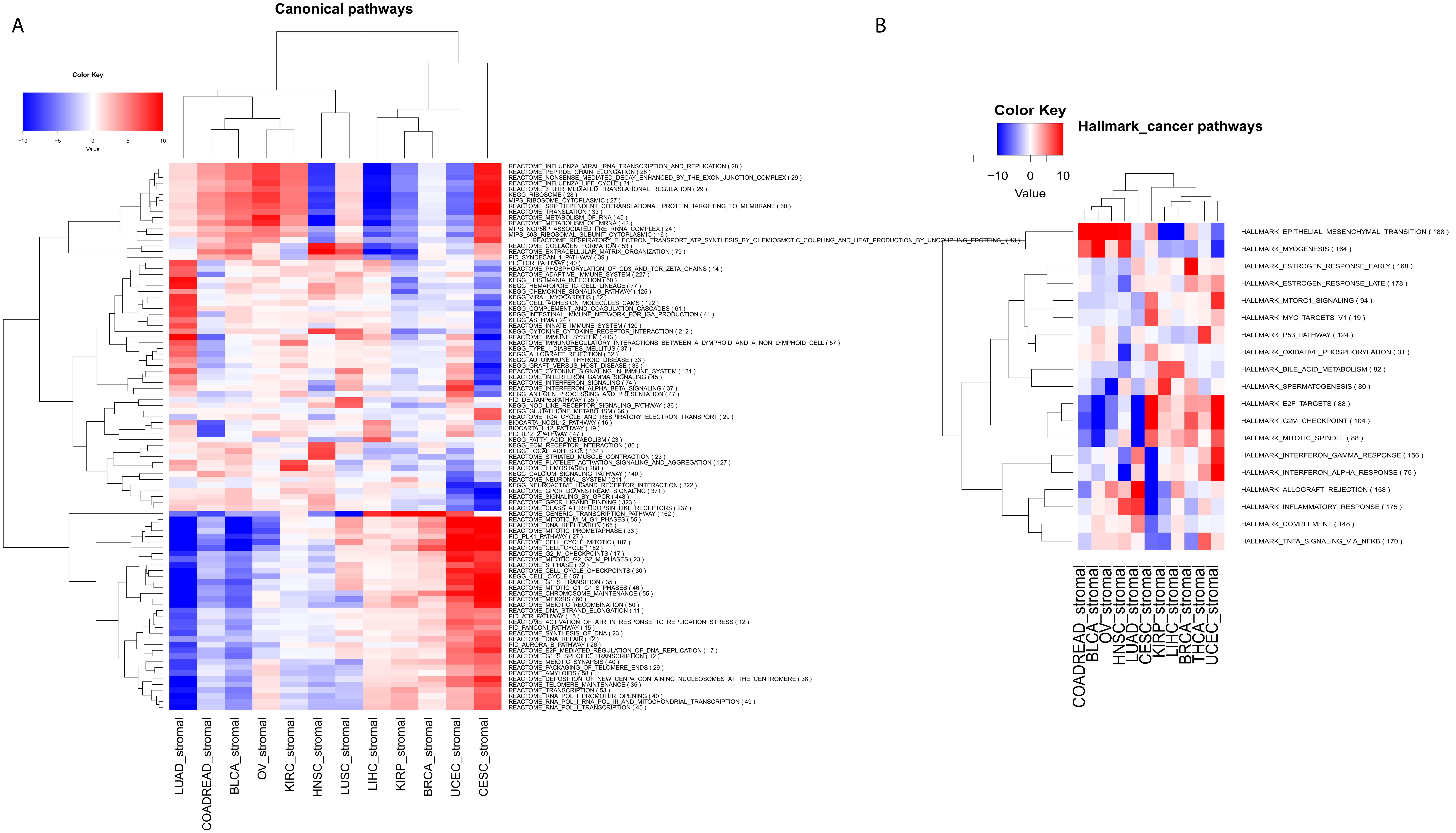

Fig. S7 Pathway analysis of stromal profiles across 13 TCGA datasets using Canonical pathways (left) or hallmark_cancer pathways (right) from MsigDB. The color indicates the significance of the up/down-regulation of that pathway in that stromal profile as compared to other stromal profiles (-log10 (p-value of Wilcoxon rank-sum test), see Method). Red color represents up-regulation and blue color represents down-regulation.

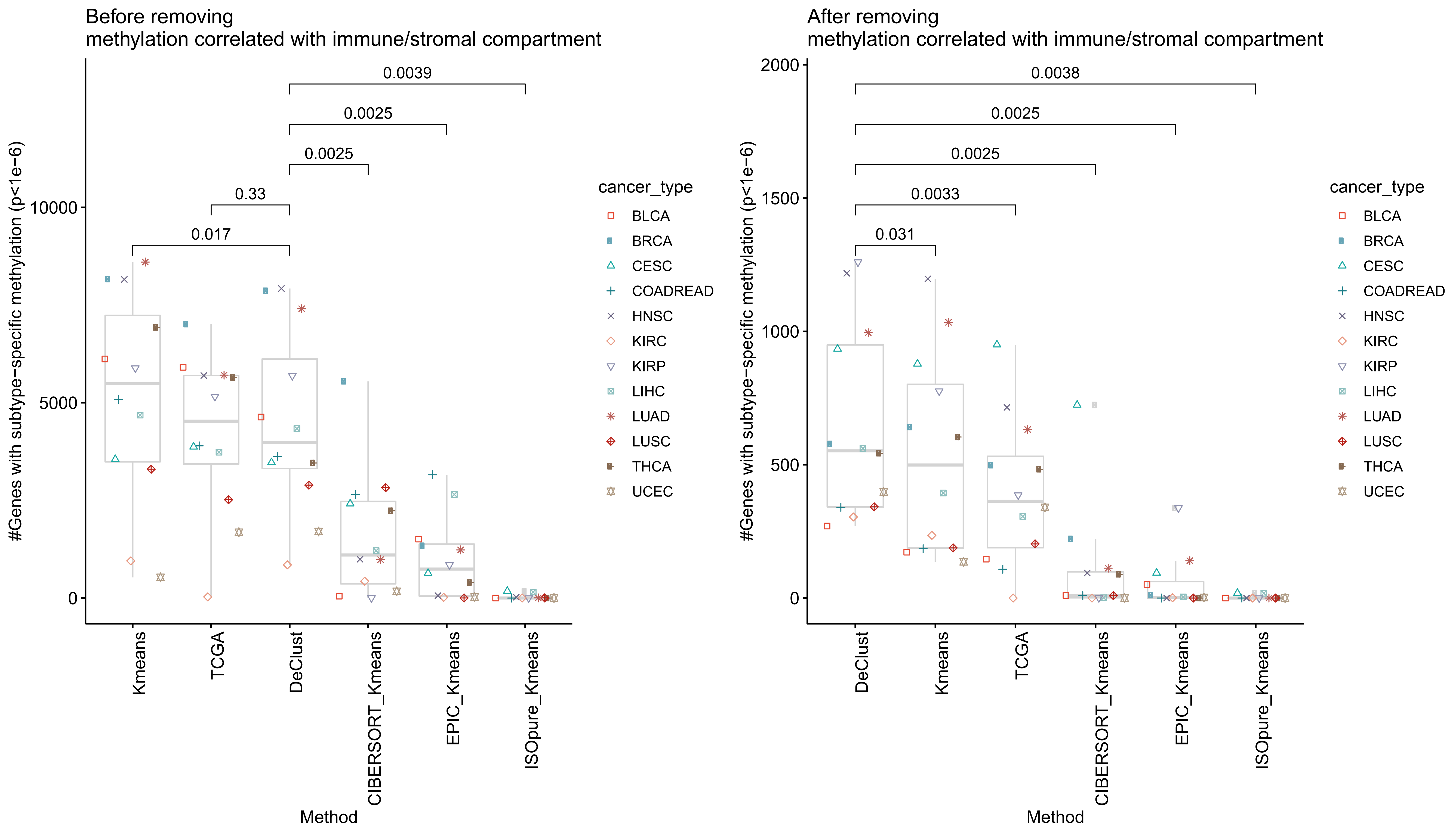

Fig. S8 Number of genes with subtype-specific methylation before (A) and after (B) methylation correlated with immune or stromal cell frequencies were removed. The methylation level of a gene was considered as subtype-specific if it is significantly associated with subtype annotation (p-value <1e-6 according to ANOVA). The methylation level of a gene was considered as correlated with immune/stromal cell frequency if it is significantly correlated with the fraction of immune/stromal cell compartment estimated by MethylCIBERSORT (p-value <0.05 according to Pearson's correlation test, we chose a more lenient cutoff here to be more inclusive).

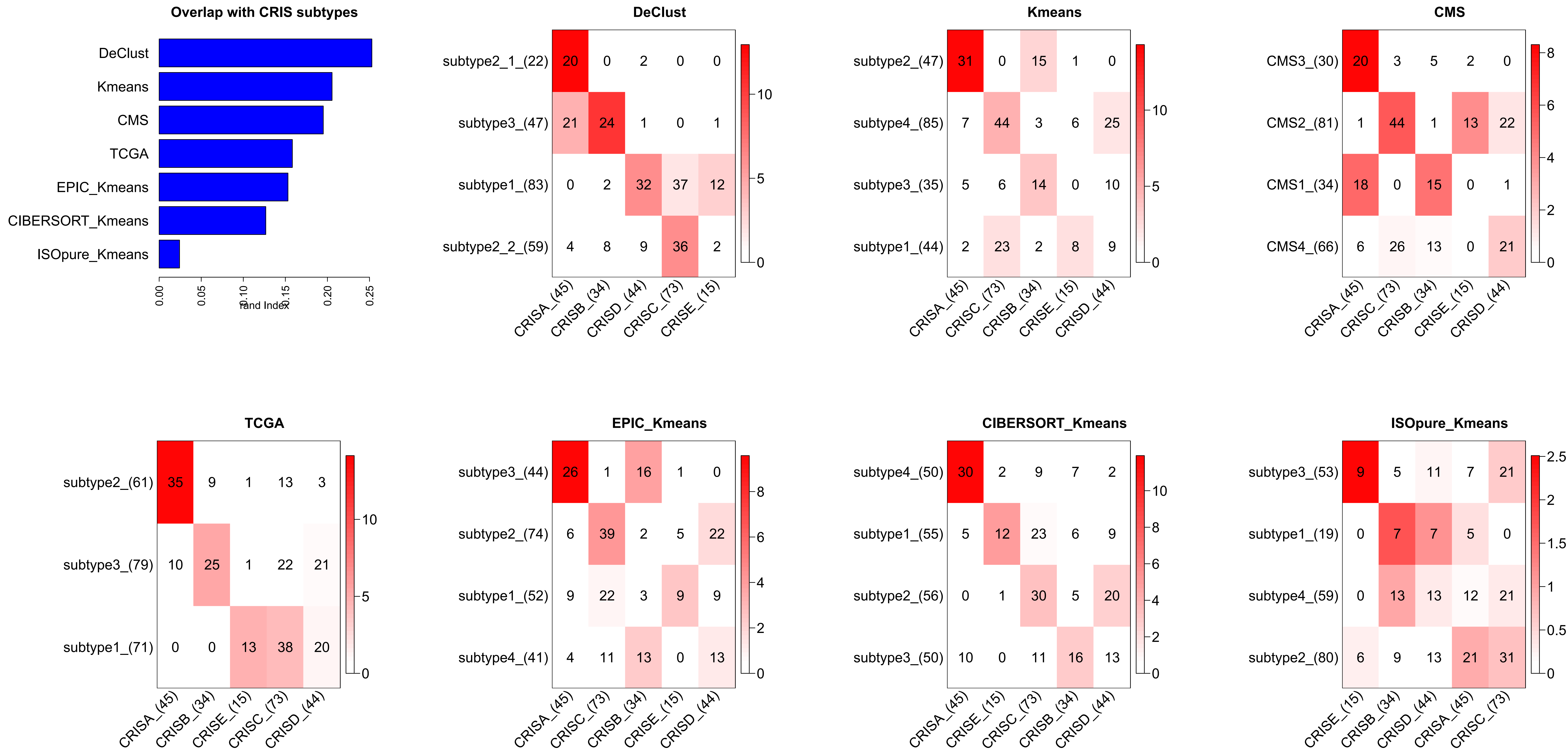

Fig. S9 Overlap between CRIS subtypes and subtypes defined by different methods. Numbers in the overlap table represent the number of samples shared by the two subtypes, and the color intensity represents the significance of the overlap.

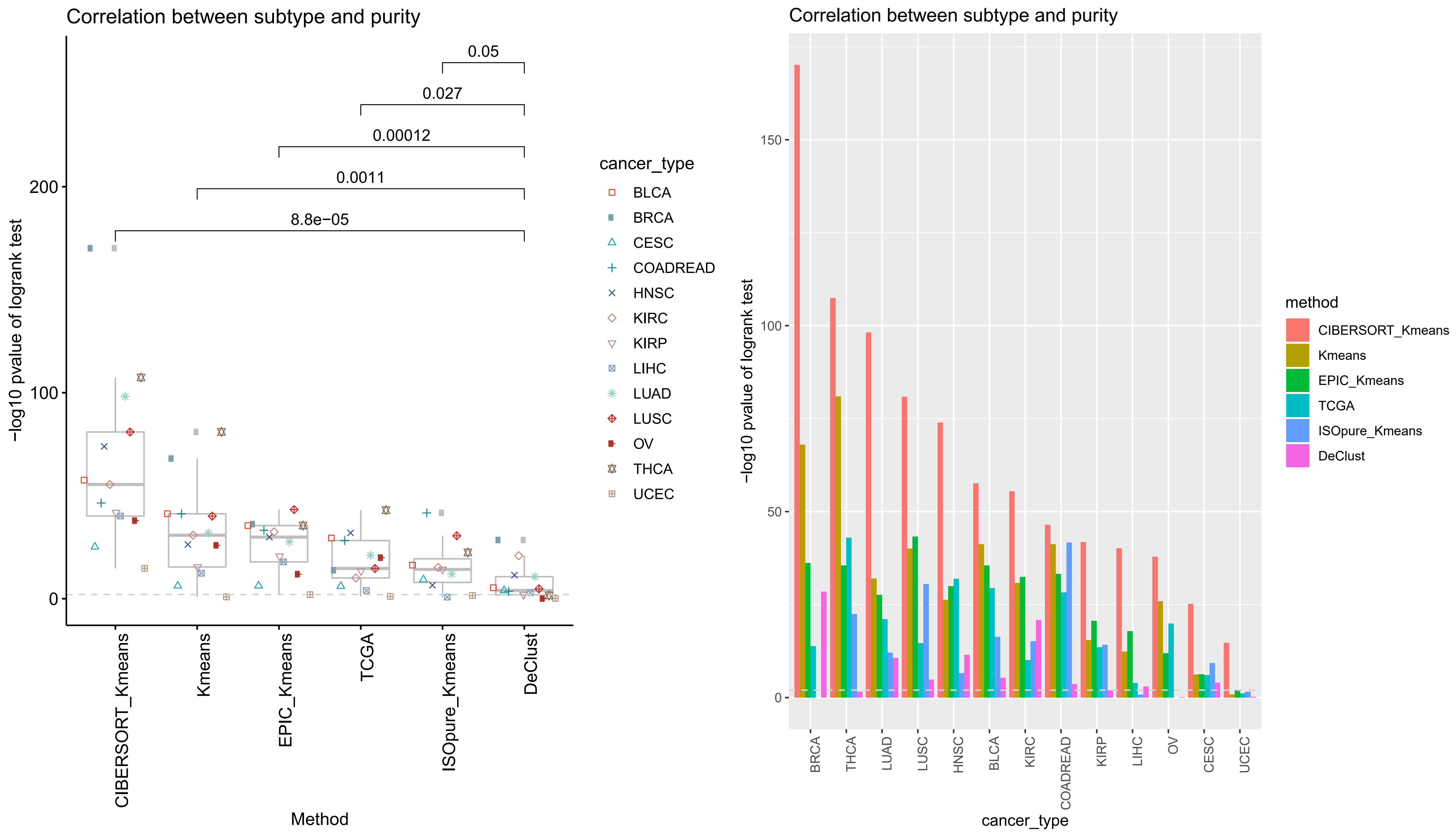

Fig. S10 Plots of the correlation between tumor purity (Consensus Purity Estimation*) and subtypes defined by different methods, grouped by methods (A) or by cancer types (B). The p-values above are the difference between DeClust and other methods according the two-sided paired t-test.

* D. Aran, M. Sirota, A. J. Butte, Systematic pan-cancer analysis of tumour purity. Nature communications 6, 8971 (2015).

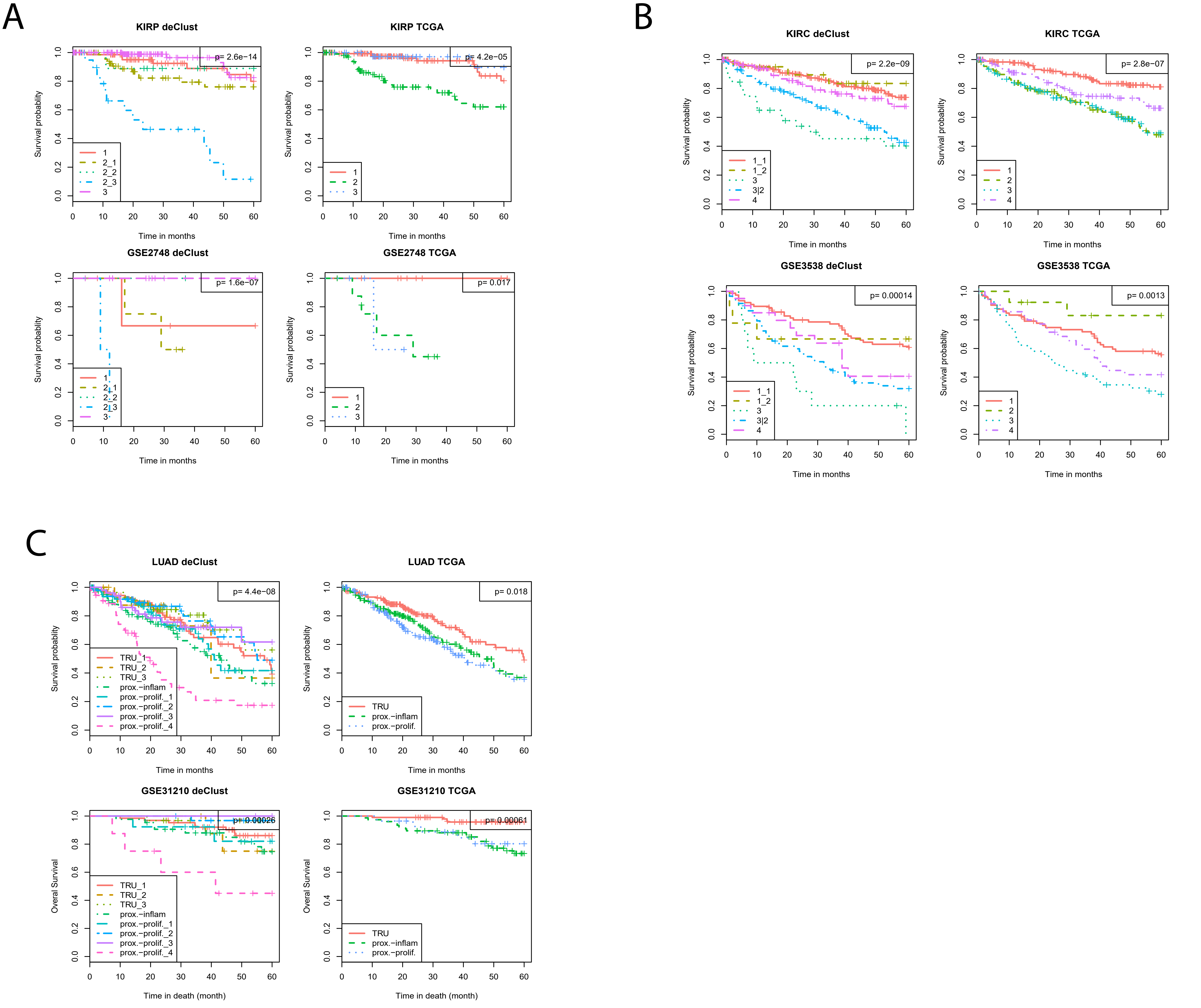

Fig. S11 Kaplan-Meier curves of DeClust subtypes (left) and TCGA subtypes (right) for TCGA dataset (top) and non-TCGA dataset (bottom) for KIRP (A), KIRC (B) and LUAD (C).

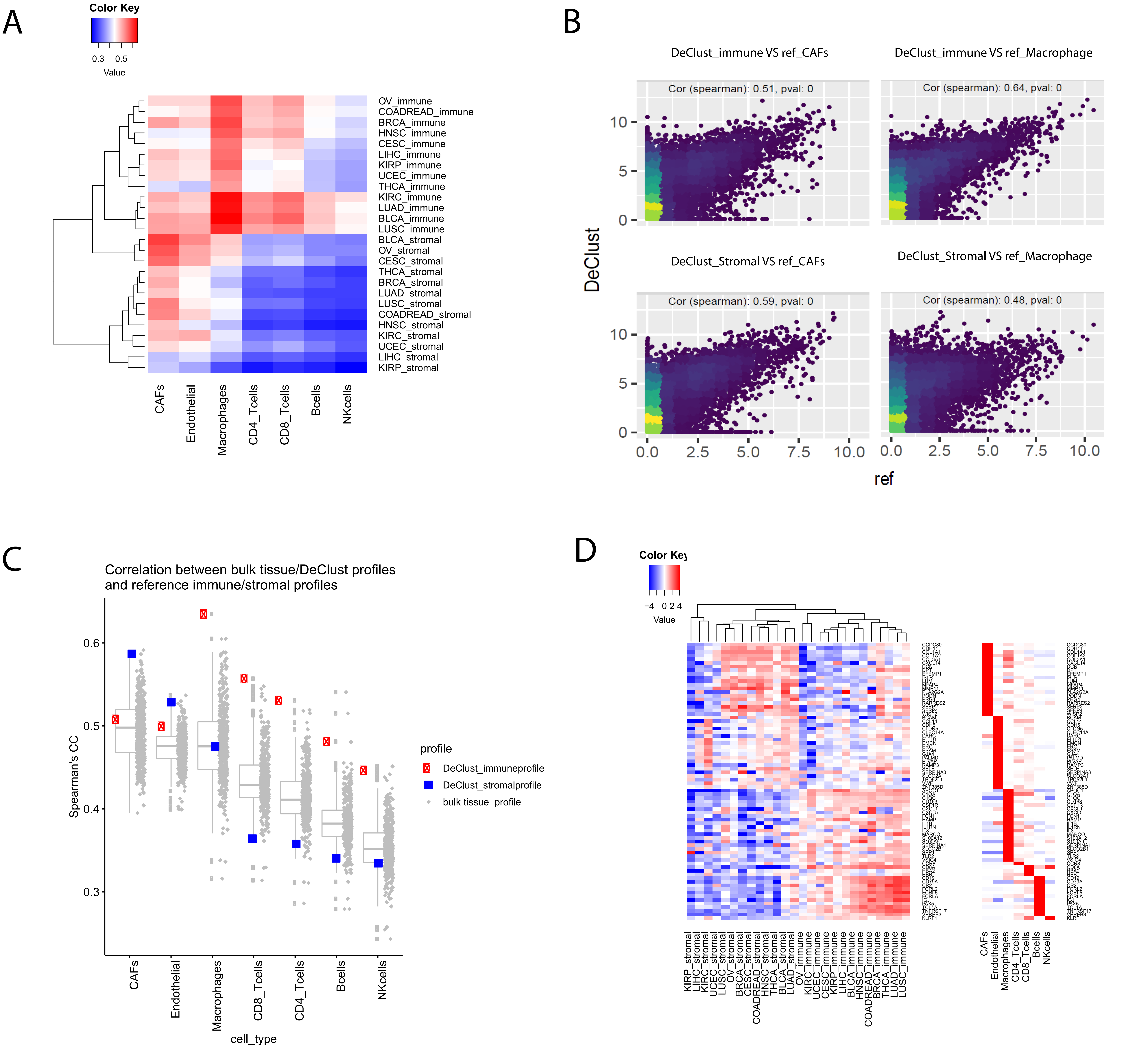

Fig. S12 (A) Spearman's correlation coefficient between immune and stromal profiles estimated by DeClust (y-axis) and reference profiles used by EPIC (x-axis). (B) Exemplary scatter plot of reference expression profile versus immune and stromal profile estimated by Declust using TCGA BLCA dataset.(C) Correlation between different reference profiles(x-axis) and bulk tissue expression profiles (grey), immune profiles (red) and stromal profiles (blue) estimated by DeClust using TCGA BLCA dataset. (D) Cell type-specific markers identified in EPIC reference profiles and their expression in immune and stromal profiles estimated by DeClust.

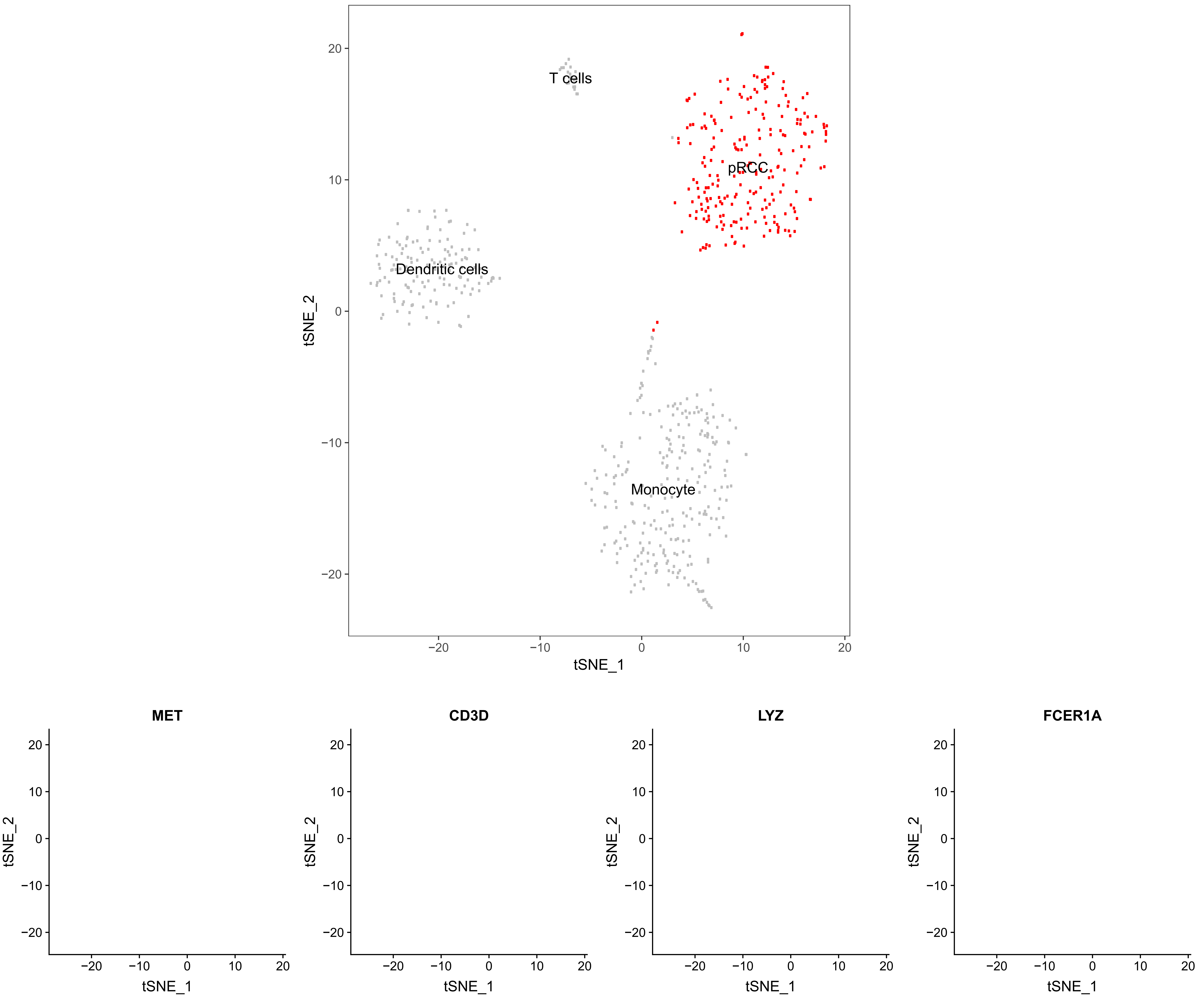

Fig. S13 t-SNE plot of scRNAseq data for pRCC samples. Cell-type specific markers used to annotate the cell clusters were shown below.

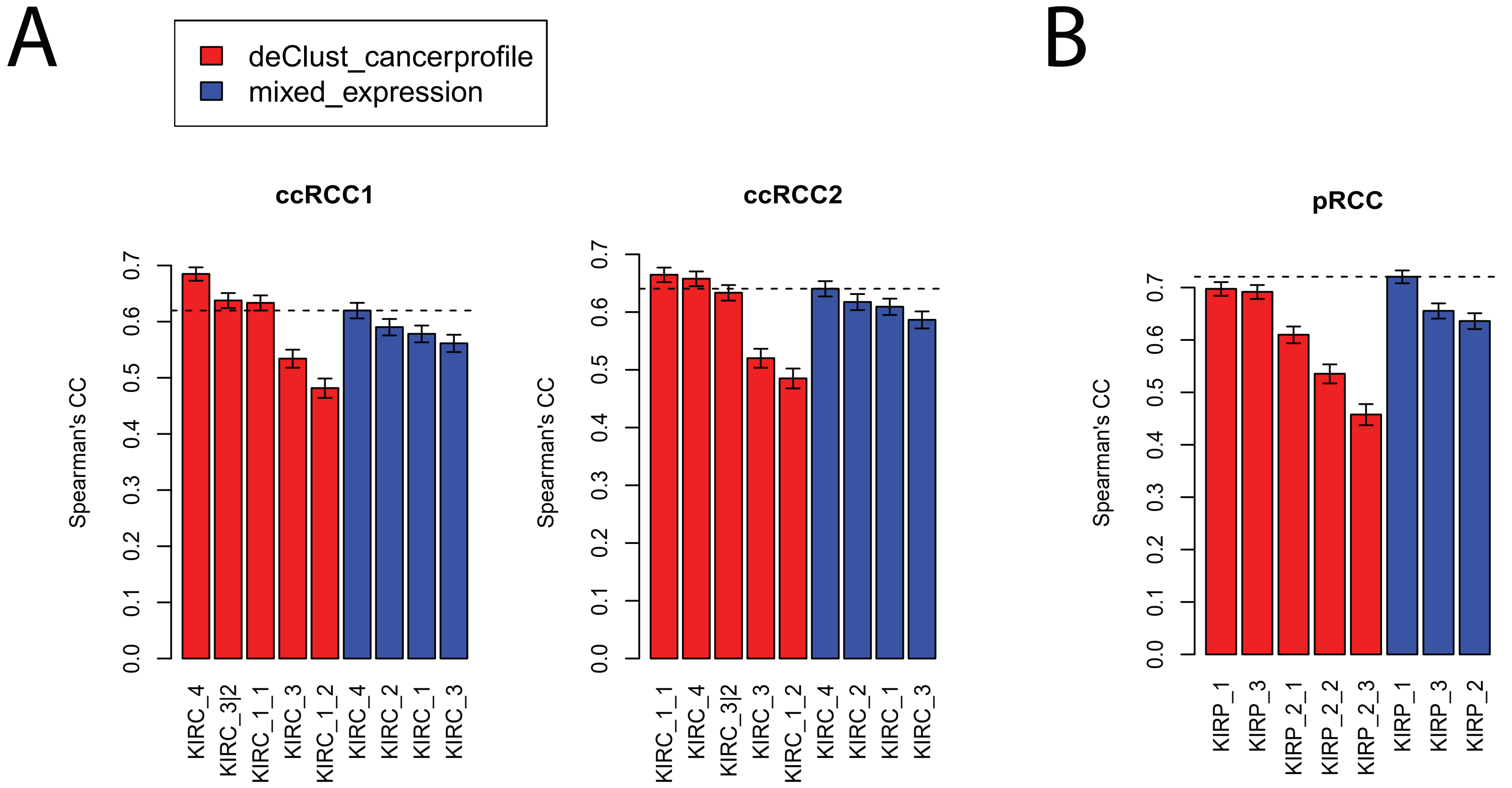

Fig. S14 Correlation between mean expression profile of each epithelial cell cluster and subtype-specific cancer profiles estimated by DeClust or by TCGA for ccRCC scRNAseq data (A) and pRCC scRNAseq data(B). Error bars indicated 95% confidence intervals.

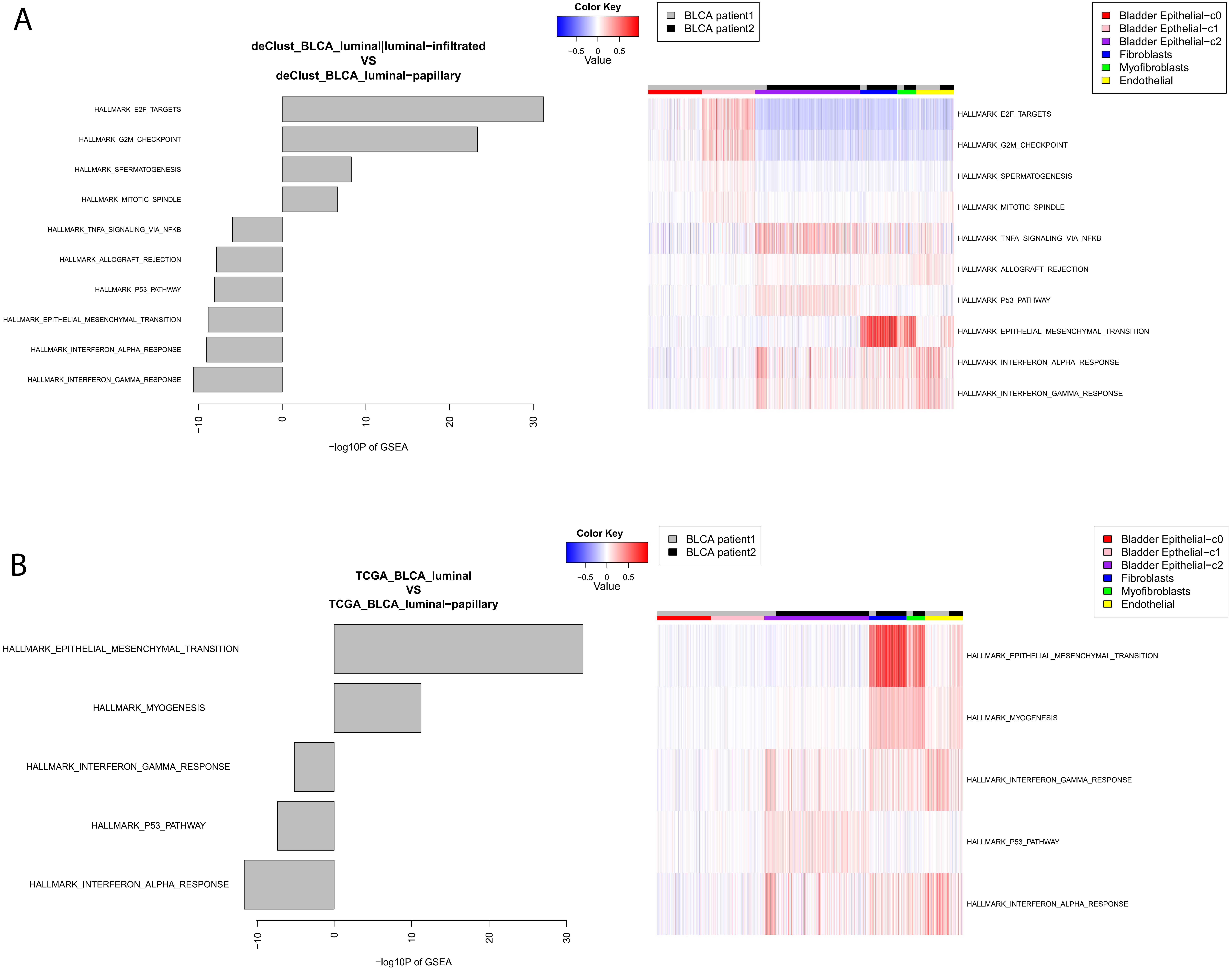

Fig. S15 Comparison of inferred cancer profiles and scRNAseq data. Bar plot of up/down-regulated pathways between two luminal subtypes defined by DeClust (A) or TCGA (B), and heat map of the expression of these pathways in scRNAseq dataset . The x-axis of bar plot represents the significance (-log10 (p-value of Wilcoxon rank-sum test)) of up/down-regulation pathways where positive indicates pathways are up-regulated in the subtype of "BLCA_luminal|luminal–infiltrated" as defined by DeClust (A) or "BLCA_luminal" as defined by TCGA (B), and negative indicates up-regulation in the subtype of "BLCA_luminal-papillary". In the heat map, color indicates the expression value (row-scaled) of these pathways in each cell calculated by averaging expression of all genes in the pathway for that cell.

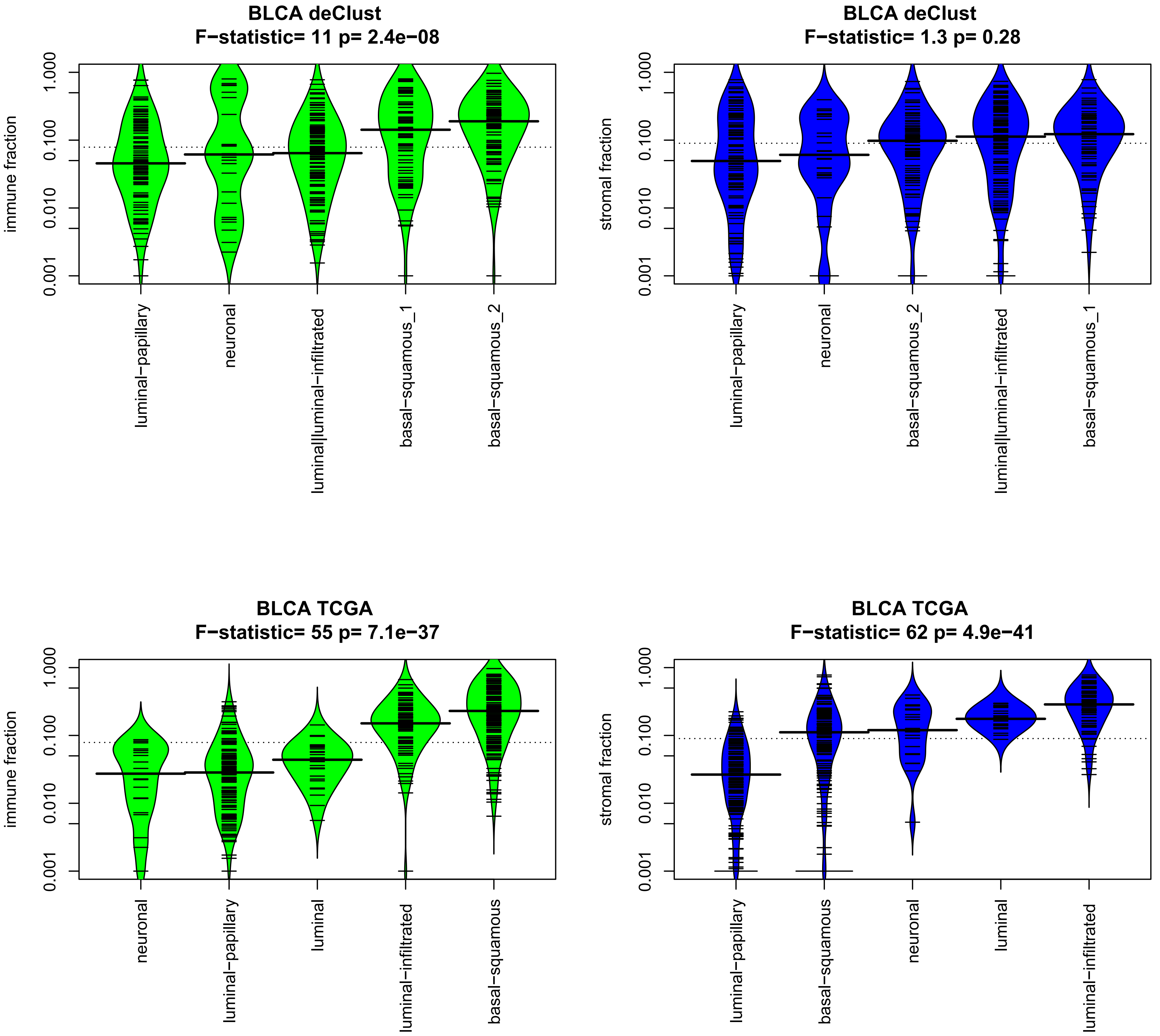

Fig. S16 Violin plot of estimated immune (left) and stromal (fraction) fractionin each subtype of BLCA. Results for subtypes defined by DeClust are at top, and by TCGA are at bottom.

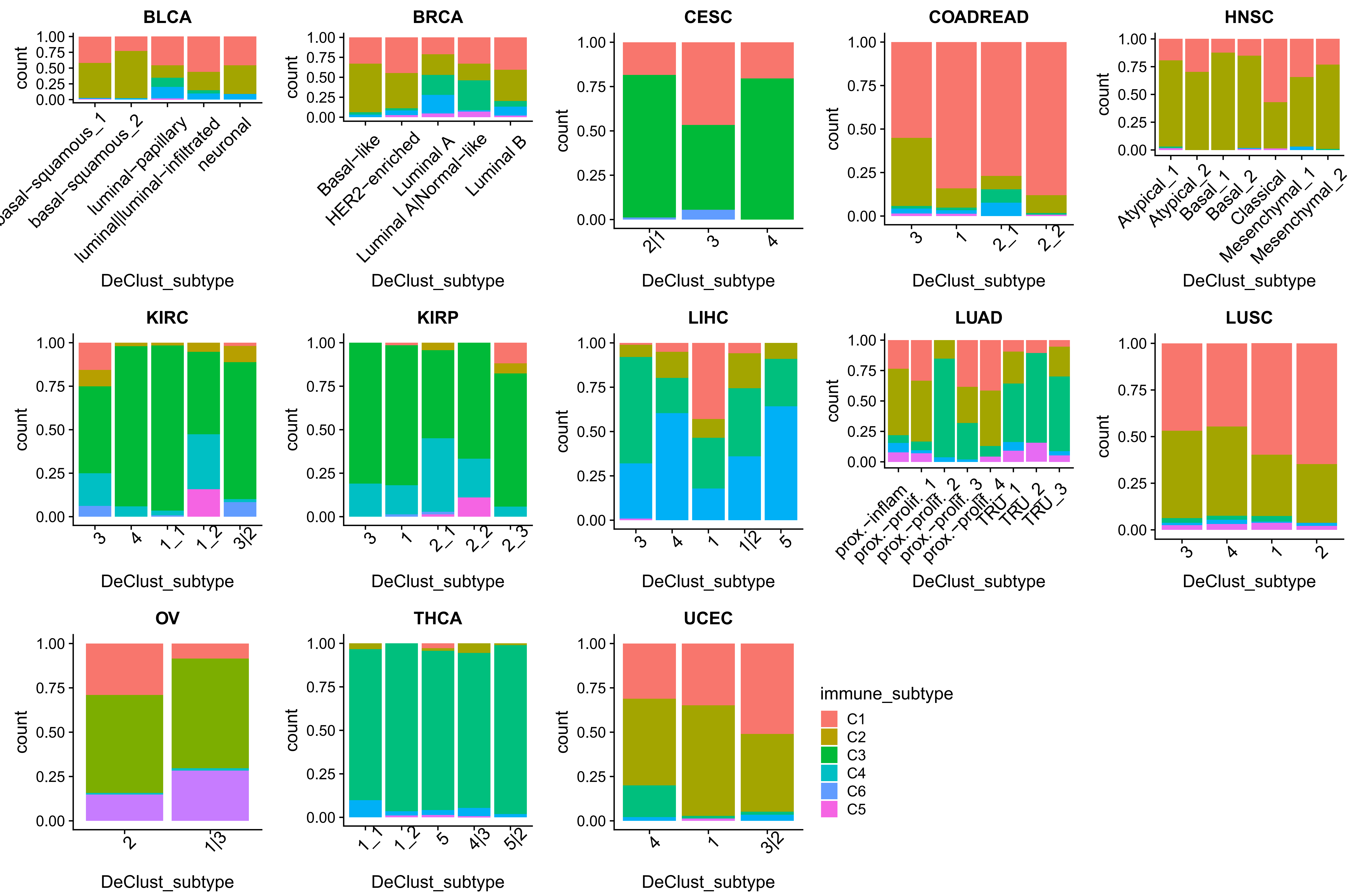

Fig. S17 Proportions of different immune subtypes within each subtypes defined by DeClust.

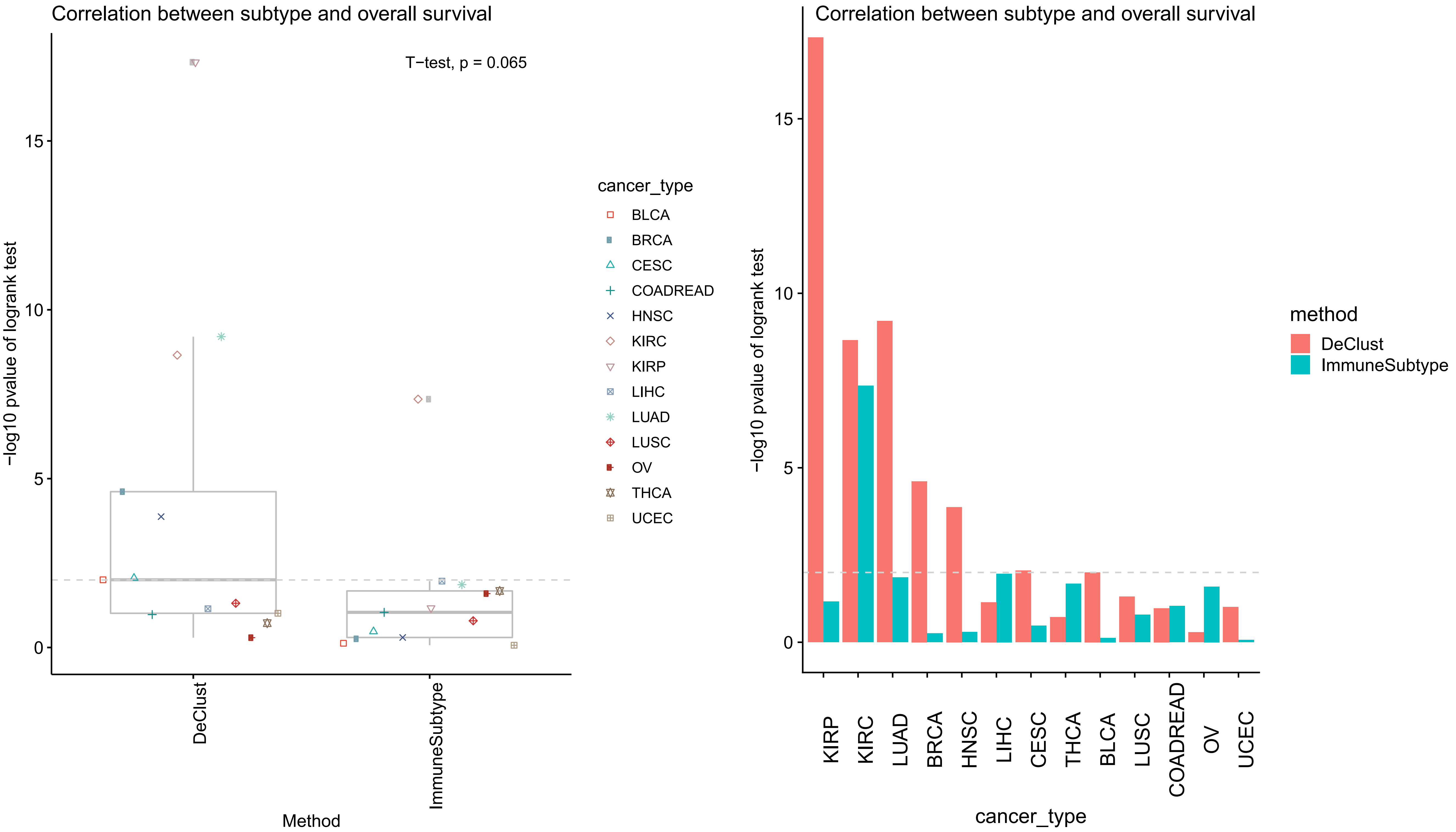

Fig. S18 Comparison of immune subtypes and subtypes defined by DeClust in association with overall survival. Plots are grouped by methods (A) and by cancer types(B)

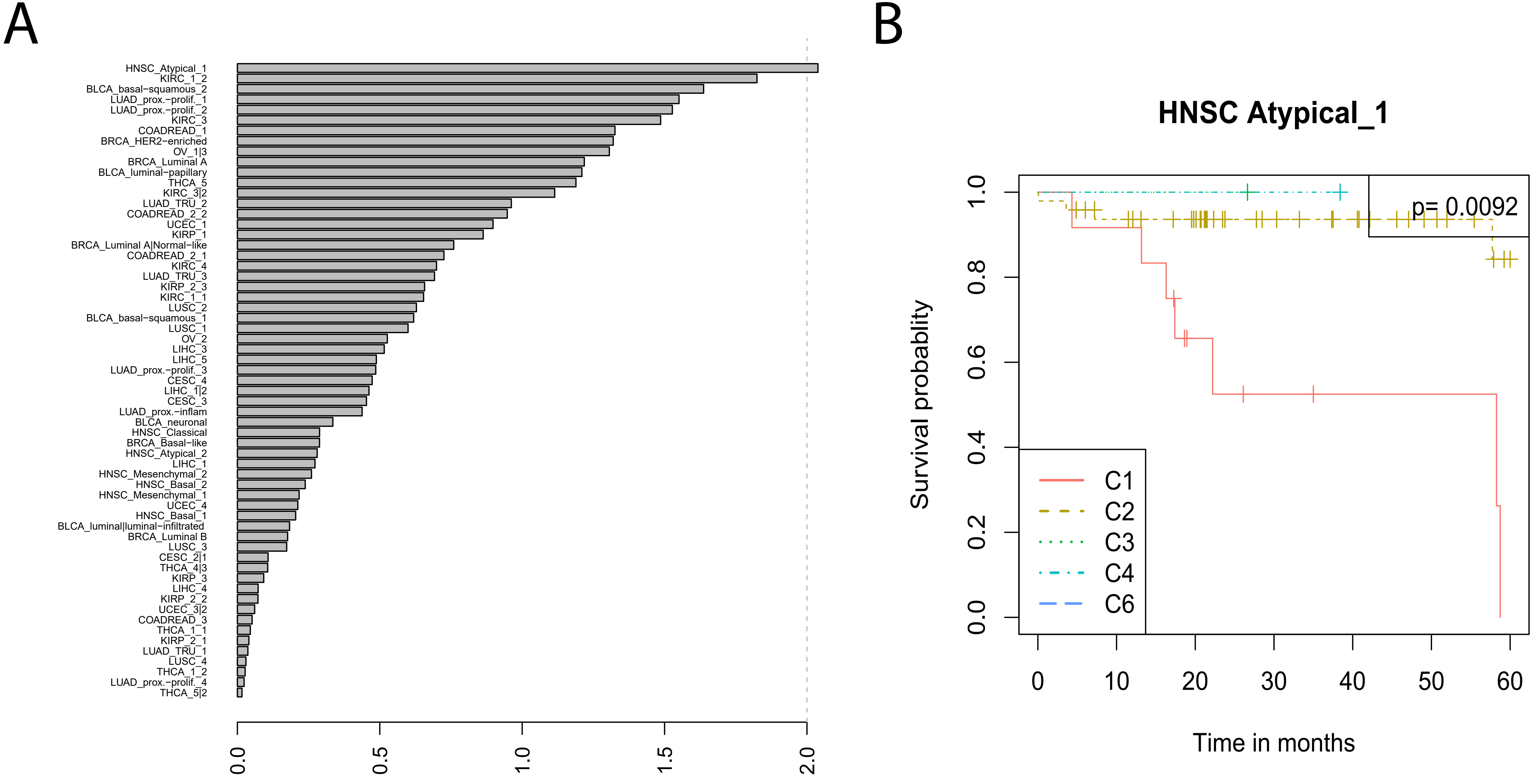

Fig. S19 (A)The association of overall survival and immune subtypes within each cancer cell-intrinsic subtype defined by Declust.(B) Kaplan-Meier curves of immune subtypes within Atypical_1 subtype of HNSC.

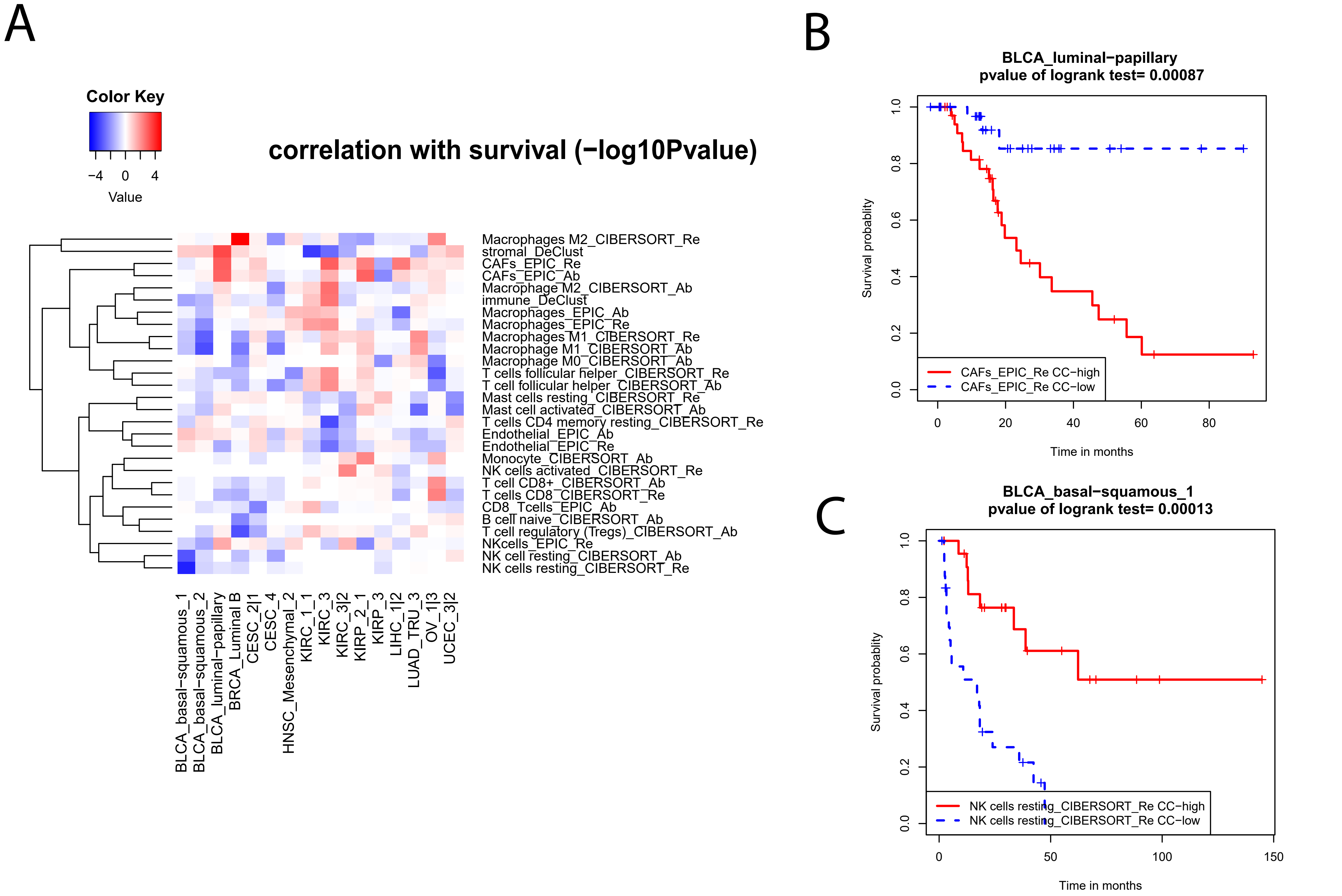

Fig. S20 (A) The association of overall survival and immune/stromal cell fraction within each subtype defined by Declust. The color in the heatmap represents the −log10(p-value) of the association (log rank test). Red means the higher cell fraction corresponds to worse survival, and blue means the opposite. Only cancer subtypes and cell fractions with at least one significant association ($p<0.05$) are shown here. The cell fractions were estimated by CIBERSORT, EPIC or DeClust, and both the absolute fraction (Ab) and relative fraction (Re) were assessed.(B) Kaplan-Meier curves of high and low CAF fraction within BLCA_luminal-papillary subtype. (C) Kaplan-Meier curves of high and low resting NK cells within BLCA_basal-squamous_1 subtype.

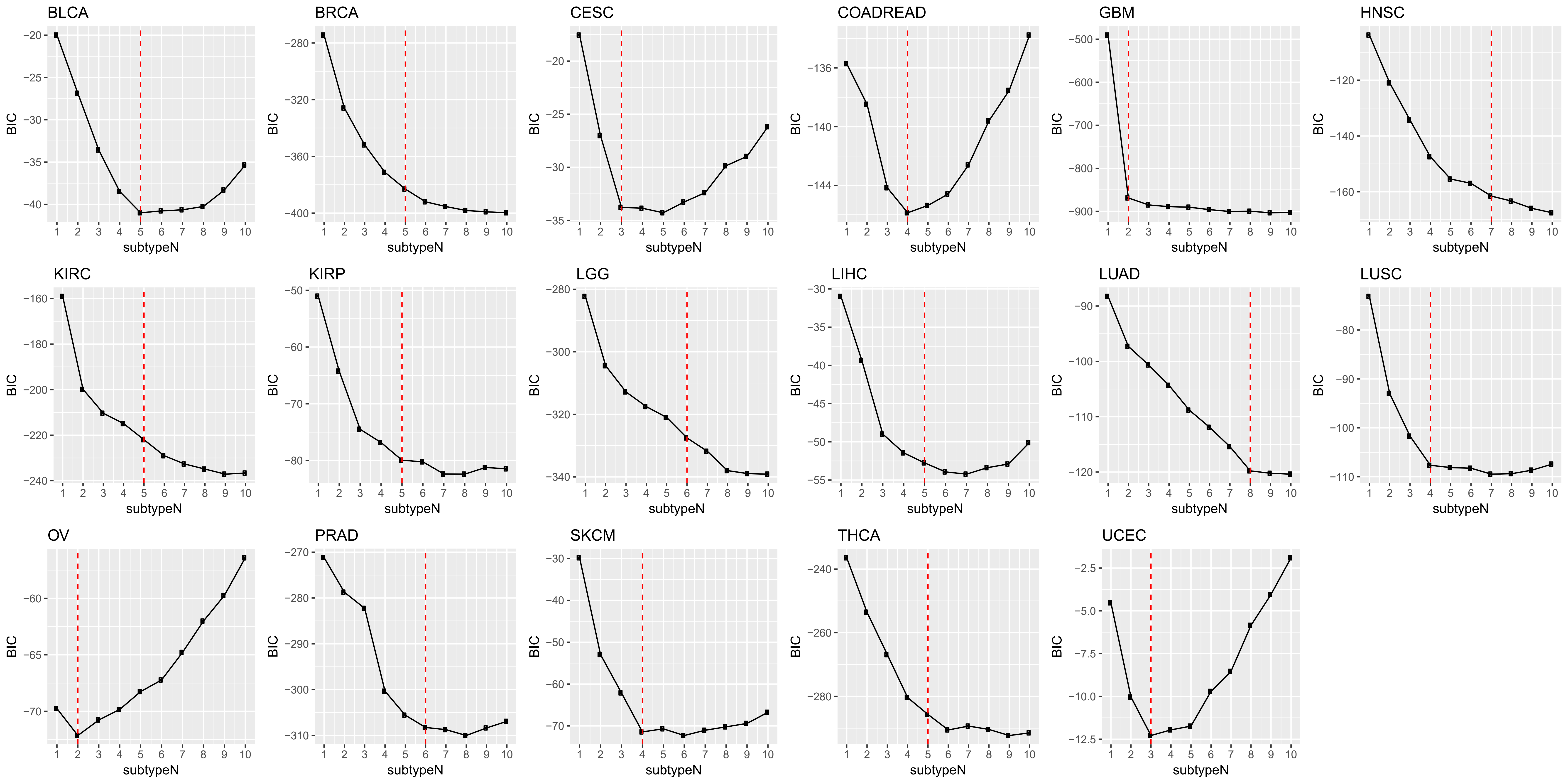

Fig S21 BICs calculated according to the DeClust model at different subtype number for each TCGA dataset. The red dotted line indicates the number of subtypes select for further analyses.

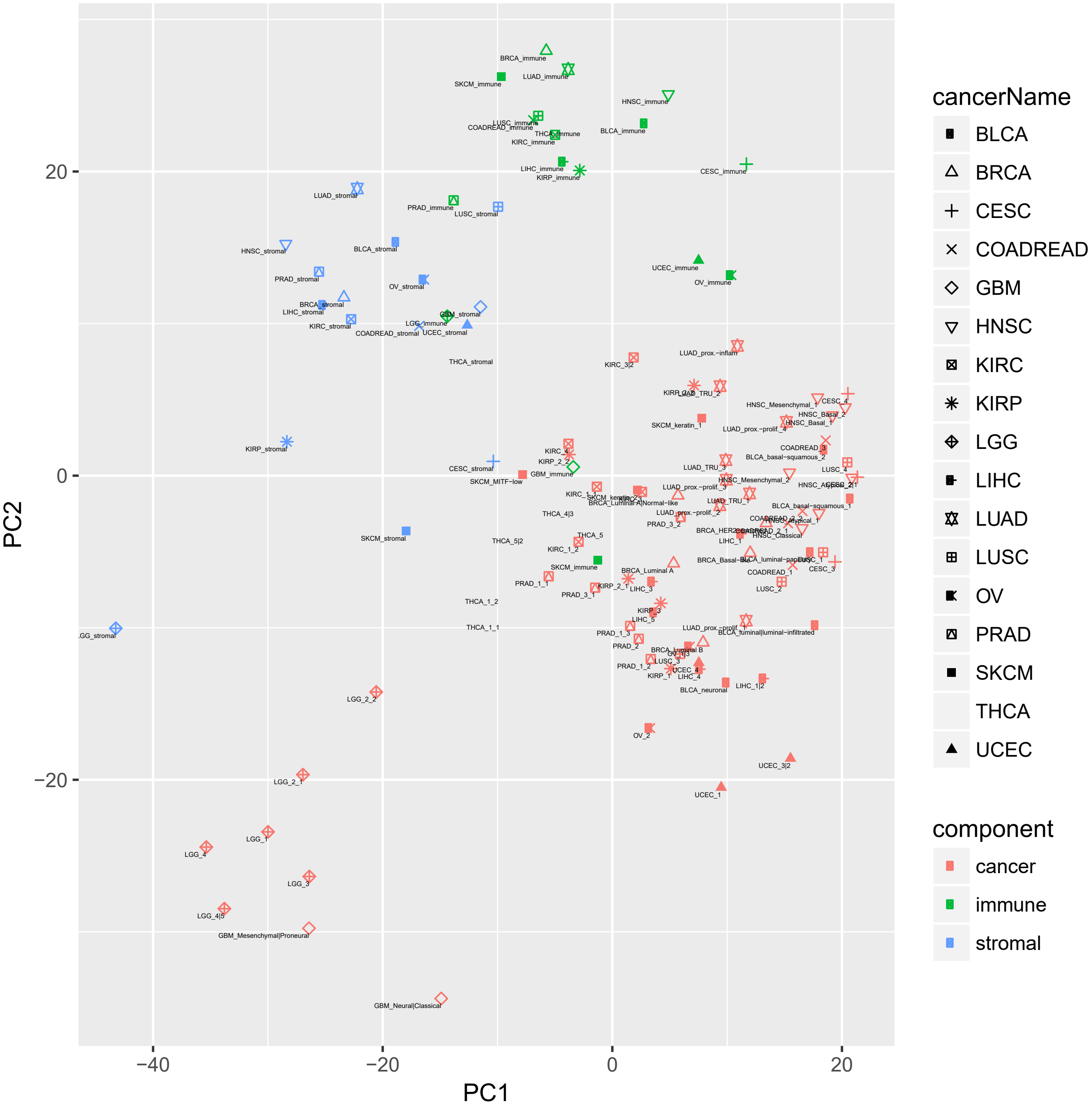

Fig. S22 PCA plot of cancer, immune, and stroma expression profiles estimated by DeClust
