# Supplementary material for: A reference profile-free deconvolution method to infer cancer cell-intrinsic subtypes and tumor-type-specific stromal profiles
Source: Genome Med. 2020 Feb 28;12:24. doi: 10.1186/s13073-020-0720-0 (PMC7049190; doi:10.1186/s13073-020-0720-0)
Supplement: Supplementary file 2 — Additional file 2 Supplementary Methods and Results, Table S1. Inputs and outputs of different methods in estimation of cell composition in bulk tumor tissues. Table S2. mapping each immune and stromal profile onto the best matching cell in human HPCA. [file 13073_2020_720_MOESM2_ESM.docx]

## Supplementary Methods and Results

## Selection of TCGA datasets

We started with 24 solid tumor datasets in TCGA([*1*](#_ENREF_1)): Adrenocortical carcinoma(ACC), Bladder urothelial carcinoma(BLCA), Breast invasive carcinoma(BRCA), Cervical and endocervical cancers(CESC), Colon adenocarcinoma(COAD), Colorectal adenocarcinoma(COADREAD), Glioblastoma multiforme(GBM), Glioma(GBMLGG), Head and Neck squamous cell carcinoma(HNSC), Kidney Chromophobe(KICH), Pan-kidney cohort(KIPAN), Kidney renal clear cell carcinoma(KIRC), Kidney renal papillary cell carcinoma(KIRP), Brain Lower Grade Glioma(LGG), Liver hepatocellular carcinoma(LIHC), Lung adenocarcinoma(LUAD), Lung squamous cell carcinoma(LUSC), Ovarian serous cystadenocarcinoma (OV), Prostate adenocarcinoma(PRAD), Rectum adenocarcinoma(READ), Skin Cutaneous Melanoma(SKCM), Thyroid carcinoma(THCA), Uterine Corpus Endometrial Carcinoma(UCEC), Uterine Carcinosarcoma(UCS). We removed 3 datasets of small sample size (n<100), i.e., ACC, KICH and UCS. COAD and READ datasets shared similar characteristics so that they analyzed together as the COADREAD dataset([*2*](#_ENREF_2)) instead of individual data sets. On the other hand, since KIRP and KIRC differed in many aspects, we analyzed the two datasets individually and removed KIPAN in our analysis. Similarly, we removed GBMLGG and analyzed GBM and LGG separately. As a result, there are 17 unique TCGA datasets left. We applied DeClust to each of the 17 datasets. The BIC curves were used to select the best number of subtypes for each dataset (Additional file 1: Fig. S21, see Methods for details).

Then, we combined the estimated expression profiles of cancer, immune, and stroma components for the 17 datasets, and investigated their relationship using principle component analysis. There were totally 113 profiles covering 10890 genes. Any missing data was imputed using k-Nearest-Neighbor method implemented in R package *impute*. The profiles were generally separated into three groups representing immune, stromal and cancer profiles, respectively (Additional file 1: Fig. S22). However, there are some outliers, such as immune profiles from PRAD, LGG, GBM and SKCM don’t cluster well with other immune profiles. We thus further mapped each immune and stromal profile onto the best matching cell in human primary cell atlas(HPCA)([*3*](#_ENREF_3)) (with highest spearman’s CC). As shown in Table S2, most immune profiles were mapped to immune cells in HPCA except for PRAD, LGG and GBM. Similarly, most stromal profiles were mapped to fibroblasts except GBM, SKCM and LGG. The tumor cell composition may be quite different for the four datasets, i.e., PRAD, SKCM, LGG and GBM, as compared to other solid tumors. PRAD is known to be immune cell deserts([*4*](#_ENREF_4)), thus there may not be enough immune cells to derive the immune cell profile for PRAD. The same arguments could apply to LGG and GBM. In addition, the main components in the stroma of brain tumors are supposed to be neuronal cells instead of fibroblasts. Similarly, the stroma of skin tumors may contain a large amount of Schwann cells([*5*](#_ENREF_5)) so that the stromal profile was mapped to Schwan cell as expected. Since these four tumor types deviated from the common constitution of other solid tumors where the three main components were cancer cells, immune cells and stromal cells (mainly fibroblasts), we thus removed the four datasets from further analysis, resulting in 13 TCGA datasets used in our analysis. Note that DeClust can be applied to tumors with unusual compositions to characterize cancer, immune, and stromal compartments. However, it is just beyond the scope of this study.

**Comparing DeClust immune/stromal profiles with commonly used reference profiles**

We compared the immune and stromal profiled inferred by DeClust for each cancer dataset with the reference expression profiles used by EPIC. The inferred DeClust stromal profiles generally showed highest correlation with reference profiles of CAFs and Endothelial cells compared with immune cells (Additional file 1: Fig. S12A). An example of scatter plot is shown in Additional file 1: Fig. S12B. Between CAFs and Endothelial, the inferred BLCA_stromal profiles correlated stronger with CAFs while the inferred KIRC_stromal profiles correlated stronger with Endothelial, consistent with Figures 3D&3E and Figure 8C&8F. DeClust immune profiles showed highest correlation with Macrophages, CD4 T cells and CD8 T cells. The inferred DeClust immune profiles also showed relatively high correlation with stromal cells. To investigate whether the high correlation were due to large batch difference between TCGA datasets and EPIC reference datasets, we compared these correlations against the “background” correlation which was the correlation before deconvolution. Taken BLCA dataset as an example, the TCGA mixed expression profiles showed much higher correlation with CAFs, endothelial and Macrophages than other immune cells (grey dots in Additional file 1: Fig. S12C). Thus, there was a general “background” of higher correlation with stromal cells. Compared to these background or mixed expression profiles (grey dots), DeClust immune profiles (red dots) consistently showed much higher correlation with immune cells, and DeClust stromal profiles (blue dots) consistently showed much higher correlation with stromal cells, indicating the effectiveness of DeClust in dissecting different compartments. Beside the above correlation analysis which considered all quantified genes, we also compared profiles using only cell type-specific signature genes, which were defined as those genes whose expression in a particular cell type was higher than the second highest cell type by more than 4 (log scale) based on EPIC reference profiles (Additional file 1: Fig. S12D, if there are more than 20 such genes for a cell type, only the top 20 were shown). As shown in Additional file 1: Fig. S12D, immune cell type-specific genes showed higher expression in DeClust immune profiles and stromal cell type-specific genes showed higher expression in DeClust stromal profiles, again demonstrating the effectiveness of DeCLust in estimation of reference profiles.

**Tumor Microenvironment heterogeneity within DeClust subtypes and their clinical relevance**

To investigate the TME heterogeneity within DeClust subtypes, we obtained sample subtyping based on immune landscape from a previous study which identified six immune subtypes across cancer tissue types([*6*](#_ENREF_6)). As shown in Additional file 1: Fig. S17, DeClust subtyping and immune subtyping was independent from each other, and there was huge immune heterogeneity within the same DeClust subtype. Although DeClust subtyping showed significantly higher association with overall survival than such immune subtyping (Additional file 1: Fig. S18), the clinical relevance of different immune subtypes within DeClust subtypes was noticeable (Additional file 1: Fig. S19). For example, within HNSC Atypical_1 subtype, immune subtype C1 was associated with significantly worse survival compared with other immune subtypes. On the other hand, when considering all HNSC patients together, immune subtypes were not associated with survival (Additional file 1: Fig. S19).

We further investigated the tumor heterogeneity within DeClust subtype based on the composition of different cell types estimated by CIBERSORT, EPIC and DeClust. For CIBERSORT and EPIC estimation, we derived both the absolute and relative fraction of each immune/stromal cell type. There were multiple significant associations between cell composition and overall survival within the DeClust subtype (Additional file 1: Fig. S20A). For example, within the luminal-papillary subtype of BLCA, a higher fraction of CAFs (estimated by EPIC) was associated with significantly worse survival outcomes (Additional file 1: Fig. S20B). Within the squamous_1 subtype, a higher fraction of resting NK cells (estimated by CIBERSORT) was associated with significantly better outcomes (Additional file 1: Fig. S20C). Thus, the clinical relevance of microenvironment could be cancer cell intrinsic subtype-specific. In summary, we observed clinical relevant heterogeneity in tumor microenvironment within DeClust subtypes, and such heterogeneity could potentially be used to further stratify patients.

## Supplementary Tables

**Table S1 Inputs and outputs of different methods in estimation of cell composition in bulk tumor tissues**

| Methods | Inputs (besides mixed tumor expression profile) | Outputs |
| --- | --- | --- |
| DeClust | Marker sets for immune and stromal compartment | Fraction of immune, stromal and cancer compartment per sample.  Reference profiles for 3 compartments.  Cancer-intrinsic subtype per sample |
| ESTIMATE | Marker sets for immune and stromal compartment | ssGSEA score for immune and stromal compartment per sample, can be transformed to cell fraction. |
| CIBERSORT (Absolute version) | Signature matrix of 22 types of immune cells (LM22) | Absolute cell fraction of 22 immune cell types per sample. |
| EPIC | Reference expression profiles for 5 types of immune cells and 2 types of stromal cells. | Cell fraction of 5 immune cell types, 2 stromal cell types and other cells per sample. |
| quanTIseq | Signature matrix of 10 types of immune cells. | Cell fraction of 10 immune cell types and other cells per sample. |
| ISOpure | Reference expression profiles for a population of normal samples | Cell fraction of cancer and non-cancer compartment per sample.  Expression profiles for cancer and non-cancer compartment per sample. |

**Table S2 mapping each immune and stromal profile onto the best matching cell in human HPCA**

| DeClust immune/stromal profile | best matching cell in HPCA | Spearman's CC |
| --- | --- | --- |
| BLCA_immune | Hs_28_DC_monocyte_derived_AEC_conditioned_d5_r2 | 0.65 |
| UCEC_immune | Hs_14_DC_monocyte_derived_mature_LPS_IFNg_48h_ | 0.61 |
| LUAD_immune | Hs_28_DC_monocyte_derived_AEC_conditioned_d5_r2 | 0.61 |
| CESC_immune | Hs_14_DC_monocyte_derived_mature_LPS_IFNg_48h_ | 0.60 |
| OV_immune | Hs_28_DC_monocyte_derived_AEC_conditioned_d5_r2 | 0.60 |
| SKCM_immune | Hs_67_BM__Prog__Pre_B_cell_CD34__r2 | 0.59 |
| BRCA_immune | Hs_28_DC_monocyte_derived_AEC_conditioned_d5_r2 | 0.59 |
| KIRC_immune | Hs_14_DC_monocyte_derived_mature_LPS_IFNg_48h_ | 0.59 |
| KIRP_immune | Hs_14_DC_monocyte_derived_mature_LPS_IFNg_48h_ | 0.58 |
| HNSC_immune | Hs_67_BM__Prog__Pre_B_cell_CD34__r1 | 0.58 |
| LUSC_immune | Hs_67_BM__Prog__Pre_B_cell_CD34__r1 | 0.57 |
| LIHC_immune | Hs_14_DC_monocyte_derived_mature_LPS_IFNg_48h_ | 0.57 |
| COADREAD_immune | Hs_14_DC_monocyte_derived_mature_LPS_IFNg_48h_ | 0.55 |
| THCA_immune | Hs_14_DC_monocyte_derived_mature_LPS_IFNg_48h_ | 0.52 |
| LGG_immune | Hs_33_Fibroblasts_breast_r2 | 0.48 |
| GBM_immune | Hs_31_Endothelial_cells_HUVEC_FPV_infected_r2 | 0.44 |
| PRAD_immune | Hs_33_Fibroblasts_breast_r5 | 0.42 |
| BLCA_stromal | Hs_33_Fibroblasts_breast_r2 | 0.65 |
| CESC_stromal | Hs_33_Fibroblasts_breast_r8 | 0.63 |
| OV_stromal | Hs_33_Fibroblasts_breast_r2 | 0.63 |
| COADREAD_stromal | Hs_33_Fibroblasts_breast_r2 | 0.60 |
| LUSC_stromal | Hs_33_Fibroblasts_breast_r8 | 0.59 |
| HNSC_stromal | Hs_33_Fibroblasts_breast_r5 | 0.53 |
| THCA_stromal | Hs_33_Fibroblasts_breast_r8 | 0.52 |
| UCEC_stromal | Hs_34_iPS_cells_iPS_skin_fibroblast_derived_SMA_3_6_r2 | 0.52 |
| LUAD_stromal | Hs_33_Fibroblasts_breast_r2 | 0.52 |
| KIRC_stromal | Hs_33_Fibroblasts_breast_r2 | 0.52 |
| BRCA_stromal | Hs_33_Fibroblasts_breast_r5 | 0.51 |
| SKCM_stromal | Hs_84_sural_nerve_Neurones_Schwann_cell_r1 | 0.50 |
| PRAD_stromal | Hs_33_Fibroblasts_breast_r2 | 0.48 |
| GBM_stromal | Hs_6_Tissue_stem_cells_BM_MSC_TGFb3_1d_r1 | 0.45 |
| KIRP_stromal | Hs_33_Fibroblasts_breast_r5 | 0.43 |
| LIHC_stromal | Hs_33_Fibroblasts_breast_r5 | 0.43 |
| LGG_stromal | Hs_64_Astrocyte_Embryonic_stem_cell_derived_d210_FGF8_specified_ | 0.34 |

## Supplementary Reference

1. K. Yoshihara *et al.*, Inferring tumour purity and stromal and immune cell admixture from expression data. *Nature communications* **4**, (2013).

2. C. G. A. Network, Comprehensive molecular characterization of human colon and rectal cancer. *Nature* **487**, 330 (2012).

3. N. A. Mabbott, J. K. Baillie, H. Brown, T. C. Freeman, D. A. Hume, An expression atlas of human primary cells: inference of gene function from coexpression networks. *BMC genomics* **14**, 632 (2013).

4. M. Bilusic, R. A. Madan, J. L. Gulley, Immunotherapy of prostate cancer: facts and hopes. *Clinical Cancer Research*, (2017).

5. Y. L. Bunimovich, A. A. Keskinov, G. V. Shurin, M. R. Shurin, Schwann cells: a new player in the tumor microenvironment. *Cancer Immunology, Immunotherapy* **66**, 959-968 (2017).

6. V. Thorsson *et al.*, The immune landscape of cancer. *Immunity* **48**, 812-830. e814 (2018).
